# QM/MM Simulations for the Broken-Symmetry Catalytic Reaction Mechanism of Human Arginase I

**DOI:** 10.1021/acsomega.2c04116

**Published:** 2022-08-30

**Authors:** Sathish Kumar Mudedla, Boyli Ghosh, Gaurao V. Dhoke, SeKyu Oh, Sangwook Wu

**Affiliations:** † PharmCADD, R&D Center, 12F, 331, Jungang-daero, Dong-gu, Busan 48792, Republic of Korea; ‡ PharmCADD, R&D Center, Workfella Business Center, Floor 5, Western Aqua Kondapur Village, Hyderabad, Telangana 500081, India; § KYNOGEN Co., Suwon 16229, Republic of Korea; ∥ Department of Physics, Pukyong National University, Busan 48513, Republic of Korea

## Abstract

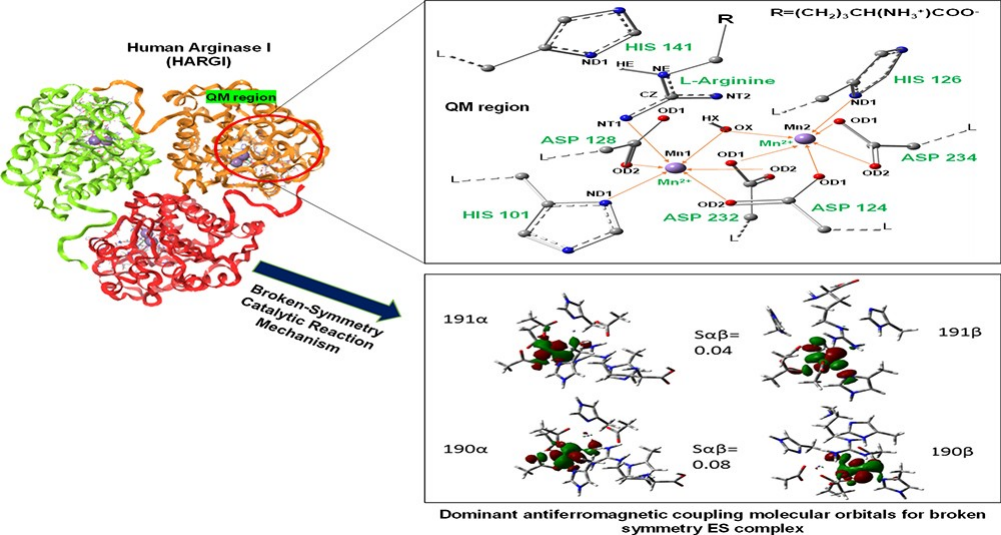

Human arginase I (HARGI) is a metalloprotein highly expressed in the liver cytosol and catalyzes the hydrolysis of l-arginine to form l-ornithine and urea. Understanding the reaction mechanism would be highly helpful to design new inhibitor molecules for HARGI as it is a target for heart- and blood-related diseases. In this study, we explored the hydrolysis reaction mechanism of HARGI with antiferromagnetic and ferromagnetic coupling between two Mn(II) ions at the catalytic site by employing molecular dynamics simulations coupled with quantum mechanics and molecular mechanics (QM/MM). The spin states, high-spin ferromagnetic couple (*S*_Mn1_ = 5/2, *S*_Mn2_ = 5/2), low-spin ferromagnetic couple (*S*_Mn1_ = 1/2, *S*_Mn2_ = 1/2), high-spin antiferromagnetic couple (*S*_Mn1_ = 5/2, *S*_Mn2_ = −5/2), and low-spin antiferromagnetic couple (*S*_Mn1_ = 1/2, *S*_Mn2_ = −1/2) are considered, and the calculated energetics for the complex of the substrate and HARGI are compared. The results show that the high-spin antiferromagnetic couple (*S*_Mn1_ = 5/2, *S*_Mn2_ = −5/2) is more stable than other spin states. The low-spin ferromagnetic and antiferromagnetic coupled states are highly unstable compared with the corresponding high-spin states. The high-spin antiferromagnetic couple (*S*_Mn1_ = 5/2, *S*_Mn2_ = −5/2) is stabilized by 0.39 kcal/mol compared with the ferromagnetic couple (*S*_Mn1_ = 5/2, *S*_Mn2_ = 5/2). The reaction mechanism is independent of spin states; however, the energetics of transition states and intermediates are more stable in the case of the high-spin antiferromagnetic couple (*S*_Mn1_ = 5/2, *S*_Mn2_ = −5/2) than the corresponding ferromagnetic state. It is evident that the calculated coupling constants are higher for antiferromagnetic states and, interestingly, superexchange coupling is found to occur between Mn(II) ions via hydroxide ions in a reactant. The hydroxide ion enhances the coupling interaction and initiates the catalytic reaction. It is also noted that the first intermediate structure where there is no superexchange coupling is similar to the known inhibitor 2(*S*)-amino-6-boronohexanoic acid.

## 1 Introduction

Human arginase I (HARGI) plays an important role in the urea cycle and catalyzes the hydrolysis of l-arginine to form l-ornithine and urea.^1^ HARGI is a metalloprotein consisting of two manganese ions in the active site, which are essential to maintain the NH_3_ detoxification rate. HARGI is a highly expressed liver cytosolic enzyme; however, extrahepatic overexpression of HARGI in the liver is linked to several disease conditions such as coronary heart disease, inflammatory bowel diseases, and pulmonary arterial hypertension.^2,3^ Therefore, extrahepatic inhibition of HARGI could be a suitable target to treat heart- and blood-related diseases without affecting its hepatic function.^4^ The available crystal structure of HARGI (PDB ID: 2AEB, resolution 1.29 Å)^5^ helps us to understand the catalytic mechanism and role of metal ions along with residues involved in the catalysis to further design new and more potent HARGI inhibitors. There are few studies reported elucidating the molecular details of the proposed catalytic mechanisms^6−8^ and the role of the two manganese(II) cations present in the arginase active site.^9−12^

The first mechanism was proposed for the hydrolysis of arginine in rat liver arginases (RARGI) and suggested a possible role of the active-site residue His141 in the catalysis.^13^ The possible role of His141 was found by the activity study of the His141Asn mutant. In the enzyme–substrate complex, the hydroxide anion, which is coordinated between both manganese(II) cations, acts as a nucleophile, whereas the positively charged guanidino group of l-arginine acts as an electrophile. In the proposed mechanism, first, a nucleophilic attack occurs on the guanidino group, followed by proton transfer from the hydroxide anion to the N_ε_ of the substrate (denoted as NE) by forming a hydrogen bond with Asp128, followed by the cleavage of the C–N bond, which leads to the release of l-ornithine and urea.

The second mechanism proposed by Khanghulov et al. was based on electron paramagnetic resonance (EPR) studies of various enzyme–inhibitor complexes for the native RARGI and its His141 to Asn mutant.^14^ In the proposed mechanism, His141 acts as a base and deprotonates the guanidino group of l-arginine. Afterward, a neutral guanidino group of l-arginine coordinates with one of the manganese(II) ions and displaces a water molecule from its coordination site. Then, a proton transfer from the water to the NE of the substrate occurs, followed by a nucleophilic attack on the carbon atom of the guanidino group, leading to the cleavage of the C–N bond and, finally, release of the products.

The third mechanism proposed by Costanzo et al. was based on the PDB crystallographic structure of ABH (2(*S*)-amino-6-boronohexanoic acid) with HARGI (PDB ID: 2AEB).^5^ Here, in the proposed mechanism, the protonation of arginine occurs at the NE position of the guanidino group by His141 instead of Asp128. Afterward, the side chain of His141 rotates 90 degrees to transfer the proton from the nitrogen atom of the imidazole ring to the NE atom. It was suggested that the roles of His141 and Asp128 residues are swapped with respect to the proposed mechanism by Kanyo.^13^

These three reported mechanisms are different in many aspects,^5,13,14^ including the protonation state of the guanidino group of the substrate, the presence of either a hydroxide ion or a water molecule as a nucleophile to form a coordination between the manganese cations, and the role of residues involved in the active site (His141 and Asp128).

Along with the afore-mentioned studies, molecular dynamics (MD) simulations^6,8,9^ and QM/MM calculations^7^ were carried out for rat liver arginase, which showed that a hydroxide anion acts as the nucleophile and attacks the substrate, and subsequently, proton transfer occurs through Asp128. Similarly, Nagagarajan et al.^11^ also proposed that a hydroxide ion acts as the nucleophile, and the interaction between the nucleophile and the substrate could lead to different initial structures of the Michaelis complex for the reaction mechanism. A recent theoretical study on HARGI, which was carried out by combining MD simulations with QM/MM, provides a full molecular understanding of the catalytic mechanism^15^ of arginases, addressing critical questions including the nature of the enzyme–substrate complex, the hydrolysis mechanism of HARGI, and the role of the manganese ions present in the active site. Also, the protonation states of arginine, active site residues, and water or hydroxide ions as nucleophiles have been studied in detail. Based on the enzyme–substrate complex produced from MD simulations, the authors have followed the mechanism postulated by Kanyo et al.^13^ Also, considering several previous reports^6−8^ that support the Kanyo et al. mechanism as a reference, the hydroxide ion was considered as a nucleophile that followed a similar mechanism in this study.

However, their calculations were restricted to the ferromagnetic state, and a total spin of manganese *S* = 5 was considered. Antiferromagnetic and ferromagnetic states can exist with high spin (5 unpaired electrons on each Mn) and low spin (1 unpaired electron on each Mn). Thus, a full understanding of the catalytic mechanism of human arginases considering both antiferromagnetic and ferromagnetic states along with different spin states (*S* = 5, 1, and 0) needs to be provided.

In this work, we used classical MD simulations coupled with a hybrid QM/MM method to understand the detailed catalytic mechanism of HARGI using different spin states and multiplicity along with both antiferromagnetic and ferromagnetic states of manganese ions. Full and detailed understanding will provide insights for designing novel efficient inhibitors for various disease conditions.

## 2 Computational Details

### 2.1 Molecular Dynamics Simulations

The protein structure of human arginase I (PDB ID: 2AEB, resolution 1.29 Å)^5^ was downloaded from the protein databank. l-Arginine was docked at the active site using Autodock Vina.^16^ The alignment of the docked ligand molecule is correlated with the inhibitor in the crystal structure. MD simulations were performed for the complex of human arginase and l-arginine with the help of the GROMACS-2020 package.^17−19^ AMBER99SB-ildn force field parameters^20^ were used for the protein, and the Lennard–Jones parameters for Mn were obtained from the literature.^21^ The structure of l-arginine was optimized using density functional theory calculations by employing the Gaussian16 package.^22^ The charges and force field parameters were generated for positively charged l-arginine and hydroxide ions using antechamber program.^23^ The positively charged l-arginine was considered as it has been shown that the complex of HARGI and positively charged l-arginine is the most adequate model to describe the catalytic mechanism of HARGI at pH 7.4. The amino acids in and around the active site were protonated similar to that in a previous report.^15^ In the active site, all aspartic acid (Asp) residues are negatively charged and histidine (His) residues have a neutral charge. The complex was solvated with TIP3P water molecules in a cubic box with a cell length of 91 Å.^24^ The total charge of the complex was neutralized by adding 0.15 nM NaCl. The solvated structures were energy-minimized using the steepest descent algorithm. The minimized structures were analyzed for equilibration at 298 K and 1 bar pressure for 1 ns by imposing position restraints on the structures. Velocity rescaling and Parrinello–Rahman algorithms were applied to control the temperature and pressure.^25−27^ Electrostatic interactions were determined using the Particle Mesh Ewald method,^28^ and bonds between hydrogen and heavy atoms were constrained with the help of the LINCS algorithm.^29^ Nonbonding interactions were calculated using a cutoff value of 12 Å. The production run was performed for 100 ns in an NPT ensemble. The structures were visualized and analyzed using Pymol.^30^

### 2.2 QM/MM Calculations

**Figure 1:**
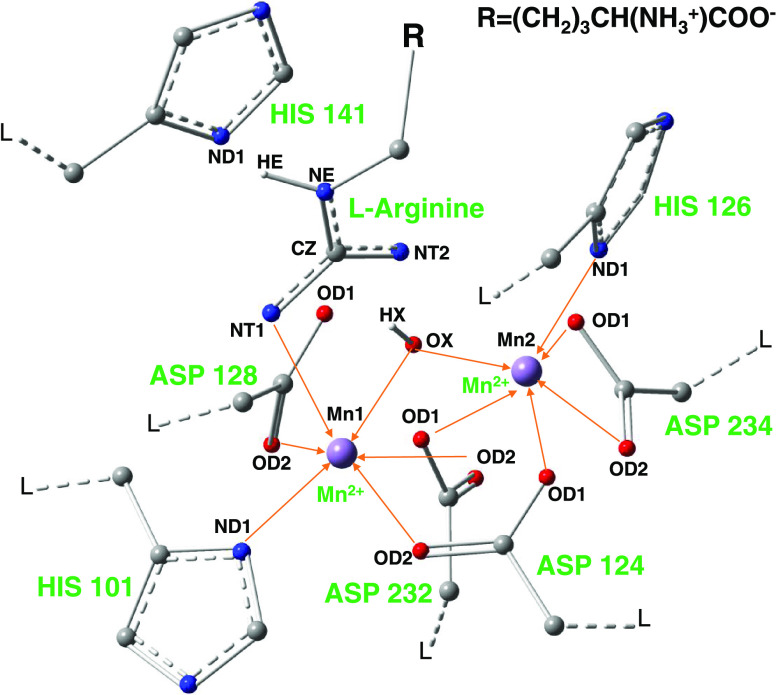
QM region used to study the catalytic mechanism of HARGI. Orange arrows represent coordination bonds with manganese cations. Residues that are considered as a part of the QM region are labeled with dark-green letters. Atoms of the substrate, nucleophile, and protein residues are also labeled in the figure. Two manganese(II) cations are labeled as Mn1 and Mn2, respectively. L represents the link atom between the QM and MM layers.

The final image from MD simulations was considered as the starting structure for QM/MM calculations to study the energetics of the catalytic mechanism of human arginase. All QM/MM calculations were performed using the ONIOM approach by employing the Gaussian16 package.^19^ The complex of arginase and the substrate is divided into QM and MM regions. The QM region included l-arginine, two Mn(II) ions, a hydroxide ion, and side chains of active site amino acids such as His101, Asp124, His126, Asp128, His141, Asp232, and Asp234 (Figure 1).

Hydrogen atoms were used as link atoms between QM and MM regions. Thus, the QM region was described using the B3PW91/6-31G* functional along with the empirical dispersion correction GD3, and the AMBER force field was used for the MM region. Based on a previous report, Mn atoms were also treated with the 6-31G* basis set. To compare the results, M062X and B3LYP-D3 functionals were employed using the 6-31G* basis set. In a previous study, the MM layer after 20 Å from the QM region was frozen, whereas in our calculations, the MM region was allowed to relax without any frozen atoms.^15^ The transition states were computed using Baker’s search, and they were confirmed with a single imaginary frequency.^31^ The reactants and intermediates were optimized to stationary points on the potential energy surface (PES), and they were confirmed with zero imaginary frequencies. We performed the intrinsic reaction coordinate (IRC) calculations for all TSs of the high-spin ferromagnetic state (HS-FS) and the high-spin antiferromagnetic state (HS-AFS) to confirm the corresponding reactants, intermediates, and products. IRC calculations showed that all transition states (TSs) were traced down to their corresponding reactants, intermediates, and products. We considered the different possible spins for ferromagnetic and antiferromagnetic states of Mn(II) ions. The broken-symmetry spin configurations were guessed using a fragment-based approach available in the Gaussview package.^32^ In this approach, each Mn and corresponding coordinate amino acids are considered as one fragment and l-arginine and the hydroxide ion are considered as individual fragments. The spin densities were calculated for all of the reactants. The charges were calculated using charges from electrostatic potentials using a grid-based method (CHELPG).^33^

The coupling parameter (*J*) value for Mn(II) ions was calculated by employing ORCA program^34^ using the following equation:
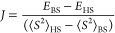
1where *E*_BS_ is the energy of the broken-symmetry state, *E*_HS_ is the energy of the high-spin state, *S*_HS_ is the total spin angular momentum of the high-spin state, and *S*_BS_ is the total spin angular momentum of the broken-symmetry state.

## 3 Results and Discussion

The main aim of this study was to provide comprehensive details about the catalytic mechanism of HARGI by considering different spin states and multiplicities along with both antiferromagnetic and ferromagnetic states of manganese ions. First, we present the data for molecular dynamics (MD) simulations, which were carried out to generate a stable enzyme–substrate (ES) complex for QM/MM calculations. Second, we explain the QM/MM optimizations for various components (reactants, TSs, intermediates (INTs), and products) involved in the catalytic mechanism of HARGI. The outcome of the QM/MM optimization at different possible ferromagnetic and antiferromagnetic states is included in the following sections.

Initially, molecular dynamics simulations were carried out to understand the stability of the enzyme–substrate (ES) complex, which is obtained from docking, and to generate the starting structure for the investigation of the catalytic reaction mechanism of HARGI. Three independent simulations were carried out for the ES complex. RMSD values for the backbone reached a plateau during the 100 ns simulation, showing (Figure S1) that the substrate is stable in the binding state. Various geometric parameters (listed in Table S1) were calculated throughout the simulations.

**Figure 2:**
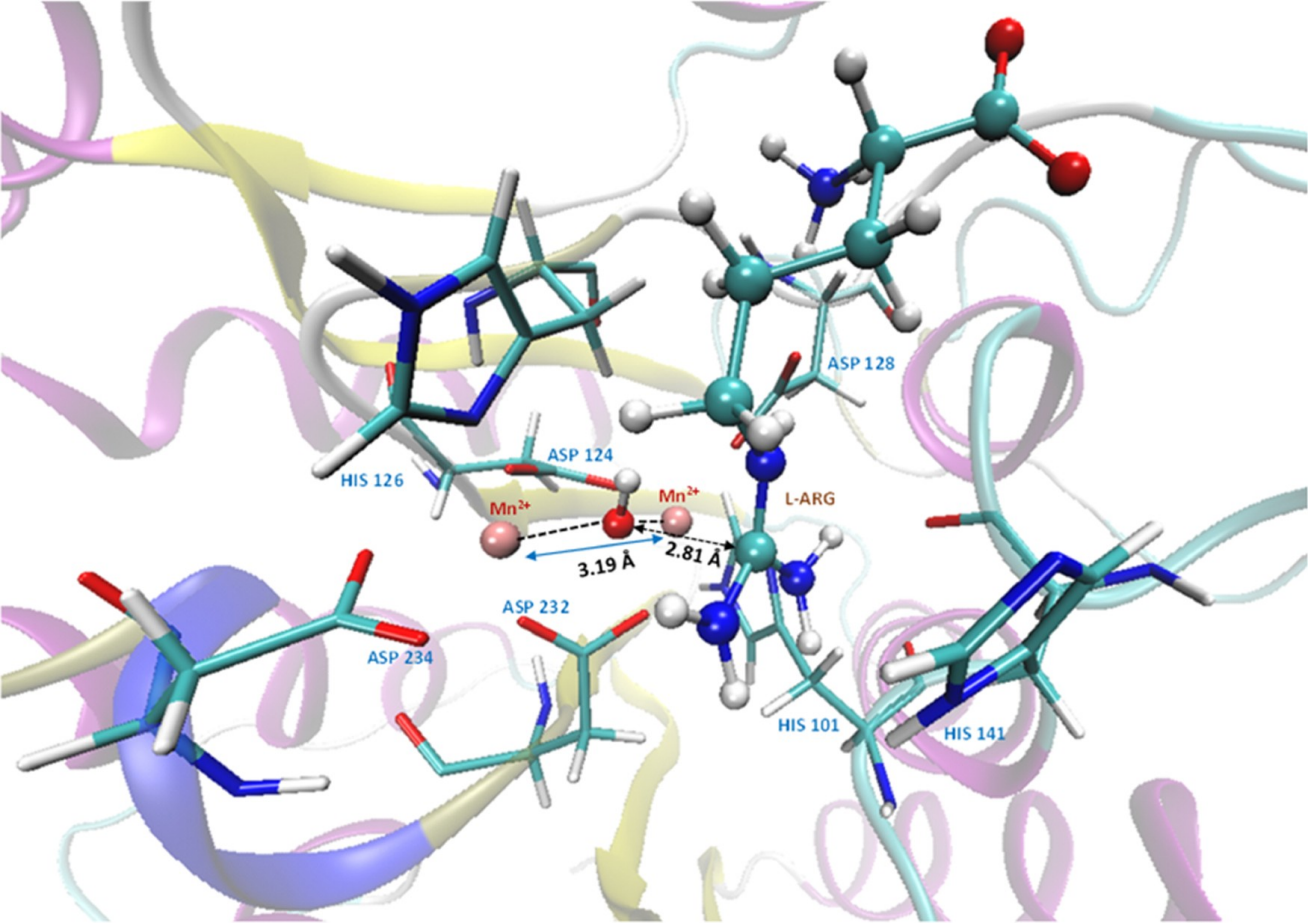
Equilibrated ES complex with the correct orientation of the substrate. The nucleophile and the substrate l-arginine are shown as a ball and sticks, respectively, whereas other active site residues are shown as sticks. The final image was extracted from the 100 ns MD simulation. The hydroxide ion was considered as a nucleophile, and the guanidino group of the substrate was treated as a positively charged species.

The calculated Mn–Mn, OX–CZ, Mn1–N1, Mn1–OX, and Mn2–OX distances are in good agreement with a previous study.^15^ During the simulations, the coordination of Mn^2+^ is maintained, as shown by the plots in Figure S1. Table S1 shows that the distance between two Mn ions is maintained along with their interactions with the hydroxide ion. Similarly, the coordinated amino acids including aspartic acid and histidine are retained throughout the simulations, as shown by the equilibrated ES complex with the correct orientation of the substrate in Figure 2.

**Figure 3:**
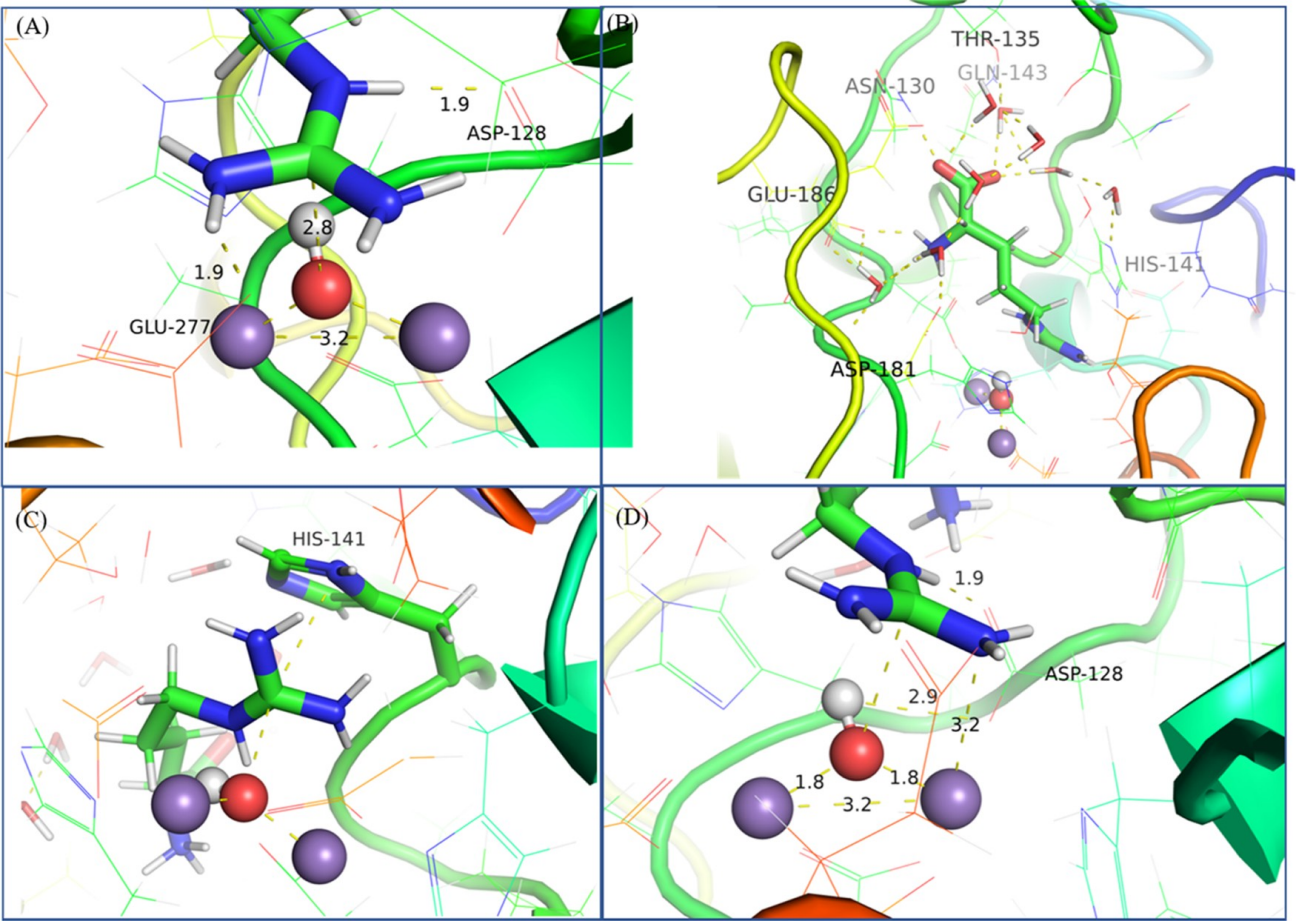
Hydrogen bond and water-mediated and stacking interactions of l-arginine with HARGI. (A) Interactions of l-arginine with the hydroxide ion, Mn(II) ions, and Asp128. (B) Water-mediated interactions of l-arginine with the surrounding amino acids. (C) Stacking interaction of His141 with l-arginine. (D) Interaction of the hydroxide ion with l-arginine, Asp128, and Mn(II) ions.

In the structure of HARGI, which was extracted from the 100 ns MD simulation, the hydroxide ion was considered as a nucleophile and the guanidino group of the substrate was treated as a positively charged species. Figures 2 and 3 show the interactions of l-arginine with the surrounding residues and water. It was found that carboxylate and ammonium groups from l-arginine also play a role in strengthening the interactions of the substrate through hydrogen bond formation with Asn130 and Glu186 (Figure 3A). Also, there are water-mediated interactions between the substrate and surrounding amino acids, which could further stabilize the substrate at the catalytic site. The carboxylate group interacts with His141, Gln143, and Thr135 through water molecules (Figure 3A). The substrate is stabilized at the catalytic site through a guanidine group via hydrogen bond interaction with Asp128 and Glu277(Figure 3B). The interaction with Glu277 keeps the substrate intact at the catalytic site. The imidazole ring in His141 has π-stacking interaction with the guanidine group (Figure 3C). It is noted that the negatively charged hydroxide ion orients toward Asp128 and forms a hydrogen bond (Figure 3D). The hydroxide ion also interacts with the carbon atom in the guanidine group, and this interaction is considered as the initial step for the catalytic reaction. All of the interactions are shown in Figure 3. It can be seen from Table S1 that the distance between OX (oxygen atom in the hydroxide ion) and CZ (carbon atom in the guanidino group from l-arginine) is within 2.5–3.2 Å. The hydroxide ion interacts with two Mn ions and holds them together within 3.3 Å distance. The presence of the hydroxide ion is crucial to attack the electrophilic site of the substrate. To understand the orientation and conformations of amino acids around the substrate in the active site of HARGI and to determine the most favorable spin state for the binuclear manganese cluster, we carried out a QM/MM calculation of the system. A full QM region consists of l-arginine, two Mn(II) ions, a hydroxide ion, and the side chains of active site amino acids such as His101, Asp124, His126, Asp128, His141, Asp232, and Asp234.

### 3.1 Probable Spin States of Human Arginase I

**Figure 4:**
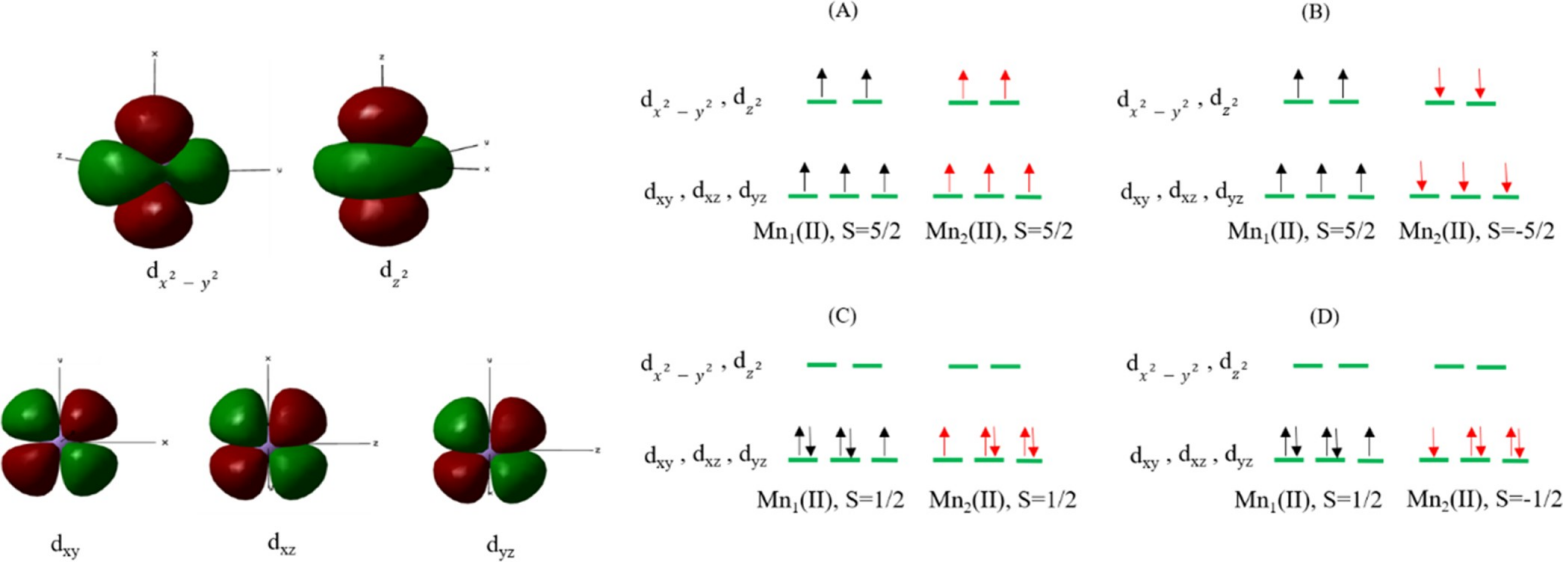
Possible spin configurations of the binuclear center of HARGI. (a) High spin: Mn_1_(II) [*S* = 5/2], Mn_2_(II) [*S* = 5/2], total spin *S* = 5, multiplicity = 11; (b) high spin: Mn_1_(II) [*S* = 5/2], Mn_2_(II) [*S* = −5/2], total spin *S* = 0, multiplicity = 1 (broken symmetry); (c) low spin: Mn_1_(II) [*S* = 1/2], Mn_2_(II) [*S* = 1/2], total spin *S* = 0, multiplicity = 1; and (d) low spin: Mn_1_(II) [*S* = 1/2], Mn_2_(II) [*S* = −1/2], total spin *S* = 0, multiplicity = 1.

All of the probable spin states of HARGI arising from the antiferromagnetic and ferromagnetic coupling of the hydroxide-ion-bridged binuclear Mn^2+^ centers have been considered in this study. The probable spin states for ferromagnetic and antiferromagnetic coupling are given in Figure 4. They include the high-spin ferromagnetic state (*S*_Mn1_ = 5/2, *S*_Mn2_ = 5/2), high-spin antiferromagnetic state (*S*_Mn1_ = 5/2, *S*_Mn2_ = −5/2), low-spin triplet state (*S* = 1/2, *S* = 1/2), and low-spin singlet state (*S* = 1/2, *S* = −1/2), which are denoted as HS-FS, HS-AFS, LS-TS, and LS-SS, respectively. The HS-FS states for both the Mn^2+^ centers (*S* = 5/2) have been considered in a previous study by Velázquez-Libera et al.^15^ Taking into account a previous study by Wu et al.,^35^ who predicted a weak antiferromagnetic coupling via a superexchange interaction between Mn(II) and Mn(II) ions in the active site of human cytosolic X-propyl aminopeptidase, we explored the mechanistic path of the broken-symmetry singlet-state HS-AFS. Initially, the ES structure is optimized in the high-spin state, where each Mn(II) ion has *S* = 5/2. The orientations of the hydroxide ion and the substrate toward the metal centers at the catalytic site are in agreement with previous studies.^15^

The ES complex of HS-AFS (Figure 4B) is more stabilized by 0.39 kcal/mol compared with that of HS-FS (Figure 4A). We used B3PW91/def2SVP, and the trend in the calculated energetics is similar to that of B3PW91/6-31G* for HS-FS and HS-AFS. We determined the energy difference between HS-AFS and HS-FS using different methods, which were 0.39, 0.43, 0.19, and 0.51 kcal/mol for B3PW91/6-31G*, B3LYP-D3/6-31G*, M062X/6-31G*, and B3PW91/def2svp, respectively. The result indicates that the antiferromagnetic state is stabilized by only below ∼0.5 kcal/mol compared with the ferromagnetic state. A previous study^15^ showed that the energy difference between the ferromagnetic and antiferromagnetic states is ∼0.2 kcal/mol. The results are in correlation with a previous study. It was found that there is no significant change in the substrate orientation toward Mn(II) ions, and the variation in the distance between two Mn(II) ions is marginal. We also considered both the ferromagnetically and antiferromagnetically coupled low-spin states of Mn^2+^ centers having multiplicities of triplet (*S* = 1/2, *S* = 1/2) and singlet (*S* = 1/2, *S* = −1/2) states, respectively, as shown in Figure 4. The ES complex for LS-TS (Figure 4C) is destabilized by ∼104 kcal/mol compared with that of HS-AFS (Figure 4B). The broken-symmetry LS-SS (Figure 4D) is also destabilized by 136 kcal/mol compared with HS-AFS. Interestingly, it was observed that the geometrical parameters changed according to the spin state of the two Mn(II) ions, as listed in Table S2. It is evident that the low-spin ferromagnetic and antiferromagnetic states are not stable compared with the high-spin states. Our QM/MM calculations show that HS-AFS is the most stable configuration among the possible spin configurations.

In addition, we also analyzed the spin density distribution to understand different spin states. The correct description of the spin density is important for understanding the electronic structures of transition metal complexes because it is related to the effective bond-order and the radical characteristics of the solutions.^36^ We calculated the Mulliken spin densities for HS-FS and HS-AFS. It can be noted from Figure S2 that Mn(II) ions have the same sign spin densities in the ferromagnetic state, whereas they have different signs in the antiferromagnetic state. However, the spin densities are similar for the proposed ferromagnetic and antiferromagnetic states of high and low spin. The spin densities of the ferromagnetic state of Mn(II) ions are +4.85 and +4.85 the spin densities of Mn1 and Mn2, respectively. For the antiferromagnetic states of Mn(II) ions, however, the values are +4.85 and −4.85, respectively.

### 3.2 Mechanism of Hydrolysis of l-Arginine by Human Arginase I

**Figure 5:**
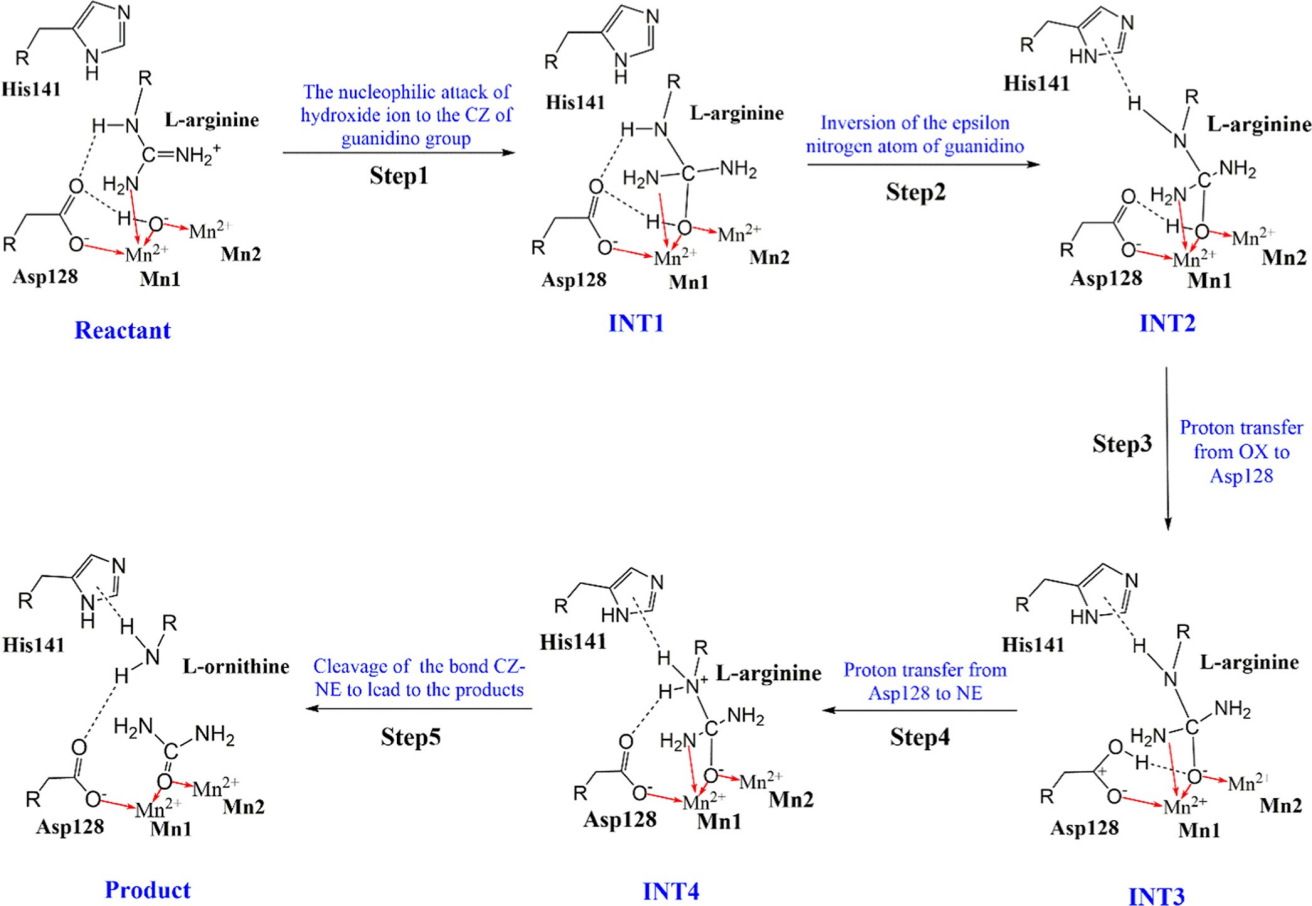
Scheme of the catalytic mechanism of HARGI.

**Figure 6:**
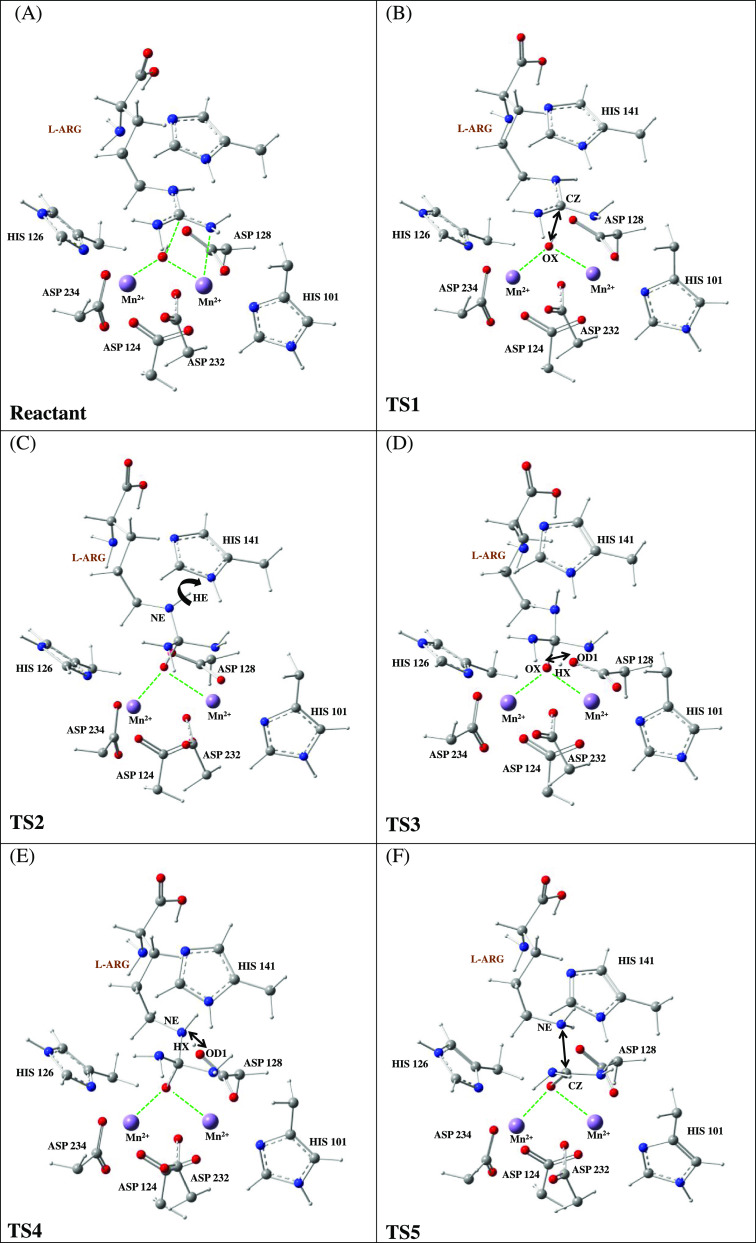
Five transition states in the potential energy profile of the mechanism of hydrolysis of human arginase. (A) Nucleophilic attack of the hydroxide ion on the carbon atom of the guanidino group of l-arginine (TS1). (B) Inversion of guanidino nitrogen and formation of the N...π interaction of the guanidino nitrogen (NE) with the imidazole ring of the histidine group, HIS-141 (TS2). (C) Proton transfer from OH to Asp128:OD1 (TS3). (D) Proton transfer from Asp128:OD1 to the nitrogen of the Guanidino group (TS4). (E) Cleavage of the C–N bond of l-arginine to form the product (TS5).

The reaction pathways for the stable high-spin ferromagnetically and antiferromagnetically coupled states were explored by QM/MM calculation, and the scheme of the mechanism that was postulated by Kanyo et al., followed by a previous report,^6−8,13,15^ is given in Figure 5. Structural analyses of the reactant shows that the path of the reaction occurring at the active site of HARGI is similar for all of the high-spin and low-spin states of the enzyme from the geometry of the ES complex. It is worth an attempt to explore the detailed reaction path for the high-spin antiferromagnetic state. The catalytic reaction of the high-spin ferromagnetic state (HS-FS) and high-spin antiferromagnetic state (HS-AFS) comprises five transition states (TSs), four intermediates (INTs), and the product. The optimized geometries of the five transition states in the reaction mechanism of hydrolysis of HARGI are shown in Figure 6. Four intermediate structures along with the product structure are shown in Figure S3.

**Figure 7:**
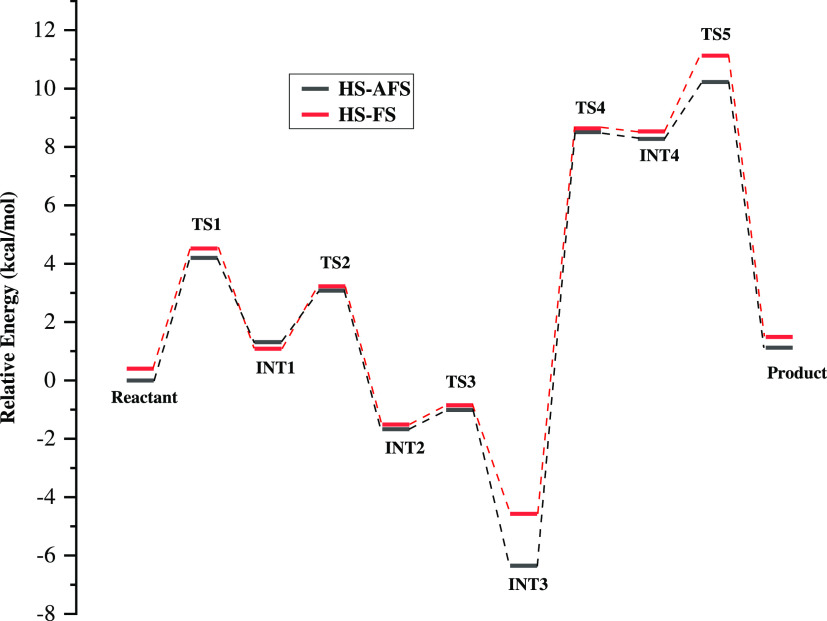
Potential energy profile for the catalytic mechanism of HARGI for the HS-AFS and HS-FS states.

The potential energy profile for the catalytic mechanism of HARGI for HS-FS and HS-AFS is shown in Figure 7. The relative energies are calculated with respect to the reactant of the HS-AFS state. Although the potential energy surfaces of the reactant, INT2, INT3, and INT4 of the HS-AFS spin state are more stabilized than that of the HS-FS state, most of the TS activation barrier of the HS-FS state is comparable to and slightly lower than that of the HS-AFS state. The energy barrier values of HS-FS are in the range of −0.85 to 11 kcal/mol. The energy barriers of HS-FS are comparable to the reported values of 3–10 kcal/mol reported in a previous study. The difference in the energy barriers may be due to the inclusion of electronic embedding in the oniom calculations and energies at a higher level of the basis set 6-311 + G**. Intermediates INT2 and INT3 are more stabilized than the reactant compared with that in a previous study. However, TS5 has a higher energy barrier similar to that reported in a previous report.^15^ The potential energy profiles (Figure S4) for HS-FS and HS-AFS at B3PW91/def2svp and B3LYP-D3/6-31G* levels of theory show that the results follow the same trend as B3PW91/6-31G*. The optimized parameters for bonds in the active site of HARGI for all of the INTs and TSs in the potential energy profile of HS-FS and HS-AFS states are listed in Tables 1 and 2, respectively.

**Table 1:** Selected Distances of All Stationary Points along the Potential Energy Surface Optimized at the UB3pw91-D3/6-31g(d) of HS State^a^

parameters	ES	TS1	INT1	TS2	INT2	TS3	INT3	TS4	INT4	TS5	product
Mn1–Mn2	3.14	3.19	3.37	3.36	3.24	3.31	3.22	3.24	3.26	3.32	3.57
OX–CZ	2.40	2.11	1.46	1.45	1.42	1.40	1.39	1.33	1.32	1.26	1.23
NT1–Mn1	2.76	2.55	2.28	2.31	2.31	2.26	2.26	2.27	2.28	2.41	2.44
ASP128:OD_1_-H*_X_*	1.70	1.67	1.48	1.43	1.03	1.27	1.03	1.28	1.44	1.90	1.92
HX–OX	0.98	0.99	1.02	1.06	1.54	1.27	1.56	2.32	2.32	2.46	3.30
HX–NE	2.74	2.59	2.43	2.50	2.62	2.50	2.62	1.23	1.12	1.02	1.02
ASP128:OD1-NE	3.39	3.15	3.10	3.33	3.26	3.25	3.25	2.49	2.54	2.85	2.88
Mn1–OX	2.02	2.06	2.36	2.39	2.41	2.25	2.28	2.22	2.24	2.37	2.14
Mn2–OX	2.03	2.06	2.27	2.29	2.29	2.33	2.26	2.13	2.13	2.17	2.29
NE–CZ	1.33	1.35	1.43	1.42	1.44	1.44	1.45	1.51	1.53	2.05	3.29

a Distances are in Å.

**Table 2:** Selected Distances of All Stationary Points Along the Potential Energy Surface Optimized at the UB3pw91-D3/6-31g(d) of HS–BS Antiferromagnetically Coupled State^a^

parameters	ES	TS1	INT1	TS2	INT2	TS3	INT3	TS4	INT4	TS5	product
Mn1–Mn2	3.16	3.25	3.37	3.40	3.39	3.32	3.26	3.24	3.27	3.33	3.58
OX–CZ	2.39	1.98	1.46	1.45	1.42	1.40	1.38	1.33	1.32	1.26	1.23
NT1–Mn1	2.74	2.54	2.28	2.28	2.28	2.26	2.26	2.27	2.28	2.41	2.44
ASP128:OD_1_-HX	1.71	1.51	1.48	1.54	1.45	1.27	1.03	1.28	1.44	1.90	1.92
HX–OX	0.98	1.00	1.02	0.99	1.04	1.27	1.58	2.32	2.32	2.46	3.30
HX–NE	2.73	2.62	2.43	2.46	2.43	2.50	2.69	1.23	1.12	1.02	1.02
ASP128:OD1–NE	3.38	3.33	2.28	3.30	3.26	3.25	3.31	2.49	2.54	2.85	2.88
Mn1–OX	2.03	2.17	2.36	2.39	2.41	2.25	2.28	2.22	2.24	2.37	2.14
Mn2–OX	2.03	2.11	2.27	2.29	2.29	2.33	2.26	2.13	2.13	2.17	2.29
NE–CZ	1.33	1.36	1.43	1.42	1.44	1.44	1.45	1.51	1.53	2.05	3.29

a Distances are in Å.

The QM/MM-optimized structure of HS-AFS has a reduced OX–CZ distance of 2.40 Å compared with that of the dynamically equilibrated structure (OX–CZ distance: 2.8 Å). The structural parameters for the active site atoms of both the HS-FS and the HS-AFS states are listed in Table 1. The hydroxide ion forms a hydrogen bond with the atom (OD1) of the carboxylate group of Asp128. The hydrogen bond distances are 1.70 and 1.71 Å for the HS-AFS and HS-FS structures, respectively.

The first step after the formation of the ES complex is the nucleophilic attack by the hydroxide anion on the carbon center (CZ) of the guanidino group of l-argininevia TS1, leading to the formation of INT1. The TS1 barriers of the HS-AFS symmetry and HS-FS states were found to be 4.19 and 4.52 kcal/mol, respectively. The stabilization energy of INT1 is 1.08 kcal/mol for HS-FS and 1.30 kcal/mol for the HS-AFS spin state. TS1 is mainly described by the formation of the OX–CZ bond between the hydroxide ion oxygen center (OX) and the guanidine carbon center (CZ). The OX–CZ distance is found to be slightly larger for the HS-AFS spin state (2.11 Å) compared with that of the HS-FS state (1.98 Å). The next step is the rotation of the nitrogen of the guanidino group via TS2, which leads to a stabilizing NH^...^π interaction of the guanidino nitrogen (NE) with the imidazole ring of His141 in INT2. TS2 has a barrier of 3.07 kcal/mol for the HS-AFS spin state and 3.22 kcal/mol for the HS-FS state. However, INT2 is more stabilized (−1.67 kcal/mol) for the broken-symmetry antiferromagnetically coupled spin state compared with that for the HS state (−1.51 kcal/mol). The NH^...^π interaction favoring proton transfer from the hydroxide anion to the carbonyl group of Asp128 via TS3 is stabilized by 0.15 kcal/mol in the case of the HS-AFS state compared with that of the HS-FS state. The proton-transferred intermediate INT3 is more stabilized (−6.34 kcal/mol) for the HS-AFS state compared with the high-spin state (−4.57 kcal/mol). The proton is subsequently transferred to the NE atom of guanidino of l-Arg via TS4 having an activation barrier of 8.5 kcal/mol for the HS-AFS state and 8.6 kcal/mol for the HS-FS state.

The NE-protonated INT4 is only slightly stabilized compared with TS4 as the NE atom of l-arginine is not a good hydrogen bond acceptor. The NE–CZ bond in INT4 is sufficiently elongated (1.53 Å) and activated to be cleaved in the next step. The NE–CZ bond is cleaved via TS5 having a barrier of 10.22 kcal/mol for the HS-AFS state and 11.12 kcal/mol for the HS-FS. Cleavage of the NE–CZ bond leads to the formation of l-ornithine and urea, which are the final products of the hydrolysis reaction of l-arginine catalyzed by HARGI. It was noted that the cleavage of the NE–CZ bond and formation of the l-ornithine product is the rate-determining step with the highest TS barriers, and this is in agreement with a previous study.^15^ Overall, it is evident that the relative energies are lowered in the case of the HS-AFS state when compared with HS-FS, and this is due to the greater stabilization of the HS-AFS reactant. Although it was confirmed from the QM/MM calculation that the HS-AFS state of HARGI is more favored compared with its HS-FS, it is difficult to predict whether only HS-AFS exists or a mixture of both the HS-AFS and HS-FS spin states of the enzyme undergoes the catalytic reaction.

### 3.3 *J*_ab_ Value and Orbital Overlap

The X-ray crystal structures of human and rat liver arginase reveal that at the bottom of the active site, a binuclear Mn(II)–Mn(II) cluster is located, and the distance between the two ions is 3.3 Å. The solvent molecule μ-hydroxide is bridged between both the spin-coupled Mn cores, and the Mn–O distance is 2.4 Å, which is in agreement with the EPR spectrum. Structures predicted by our QM/MM calculation correspond well with the crystal structure and EPR-predicted geometric parameters (Tables 1 and 2). The EPR measurement in the wild-type arginase predicted antiferromagnetic coupling between the Mn ions, with the exchange coupling constant *J*_ab_ = ∼ −2.0 cm^–1^.^14,37^ Dismukes et al. and Saito et al.^7^ suggested a possible bridging of μ-aqua in place of μ-hydroxide based on the weaker magnetic coupling constant (*J*_ab_) calculated and predicted by EPR studies. However, Christianson et al.^5^ proposed that the catalytic hydrolysis reaction in HARGI is initiated by the attack of the substrate by the metal-activated bridging hydroxide ion.

**Figure 8:**
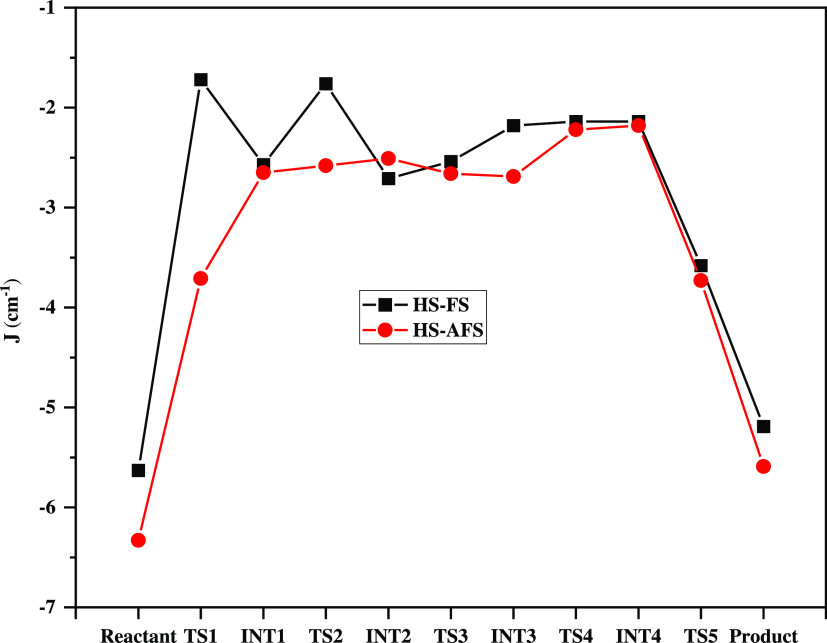
Calculated *J*_ab_ along the catalytic hydrolysis pathway by HARGI.

We calculated the Mulliken spin population on the Mn cores for the high-spin and high-spin BS-optimized intermediates and transition states all along the potential energy surface. The results confirm that the spin (*S* = 5/2) on the Mn1 and Mn2 centers are ferromagnetically and antiferromagnetically coupled in the HS-FS and HS-AFS states in all of the reaction species. In the next step, to measure and analyze the ferromagnetic and antiferromagnetic coupling between two local spins on each Mn(II), the exchange coupling constants were calculated for all of the intermediates and TSs along the PES for both HS-FS and HS-AFS optimized structures, and they are presented in Figure 8.

*J*_ab_ values are high for HS-AFS when compared with HS-FS throughout the reaction path, except in the case of INT2. This emphasizes that a strong coupling exists between the two Mn(II) ions in the case of BS compared with the HS state. It can be noted from Tables 1 and 2 that Mn(II)–Mn(II) distances are slightly lower compared with that of the HS-FS state. The decrease in the Mn(II)–Mn(II) distance increases the coupling between them, which also reflects the *J*_ab_ values. However, the calculated coupling values for the reactant (−5.6 cm^–1^ and −6.33) are not in agreement with the experimental values (∼ −2.0 cm^–1^).^37^ This is because the experimental values obtained for arginase with borate are similar to those of the intermediates (INT1, INT2, INT3, and INT4), where the hydroxide ion is transferred to the carbon in the substrate. Hence, *J*_ab_ values of intermediates in the reaction are in agreement with experimental values for both HS-AFS and HS-FS states. Also, the presence of the hydroxide ion increases the coupling between two Mn(II) ions by holding them together. In our study, comparing the *J*_ab_ values for all of the intermediates and TSs along the catalytic pathway, it was noticed that a decrease in the *J*_ab_ value occurs due to the nucleophilic attack of l-arginine on the hydroxide ion in TS1 (*J*_ab_ = −1.7 cm^–1^ in HS-FS and −3.7 cm^–1^ in HS-AFS). This change in the value of *J*_ab_ indicates a reduction in the magnetic interaction in the Mn–O–Mn core when the bridging hydroxide ion is transferred to the carbon center of the guanidino group of l-arginine. This decreased value of *J*_ab_ remains almost unvaried all along the pathway.

An increased *J*_ab_ was observed in the product where there is no bridging between the Mn^2+^ cores. To analyze the change in the *J*_ab_ value, the orbital overlap between the magnetic molecular orbitals (*S*_αβ_) was calculated for all of the intermediates and TSs along the pathway, and the results are listed in Table 3.

**Table 3:** *S*_αβ_ Values for the Overlapping Molecular Orbitals

	molecular orbital-*S*_αβ_ value				
species	190(α/β)	191(α/β)	192(α/β)	193(α/β)	194(α/β)
ES	0.08	0.04	0.03	0.02	0.01
TS1	0.05	0.03	0.02	0.01	0.003
INT1	0.04	0.03	0.02	0.01	0.005
TS2	0.03	0.03	0.02	0.01	0.001
INT2	0.05	0.03	0.02	0.01	0.005
TS3	0.04	0.03	0.02	0.01	0.008
INT3	0.05	0.03	0.02	0.01	0.01
TS4	0.04	0.03	0.02	0.02	0.01
INT4	0.04	0.04	0.02	0.01	0.01
TS5	0.05	0.04	0.02	0.01	0.003
product	0.05	0.04	0.02	0.01	0.003

The atomic orbital overlap matrix *S*_αβ_ is defined by the equation
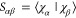
2where {χ_τ_|τ = 1,2,...,*m*} is a collection of atomic orbitals located on the different atomic centers in the molecule.

**Table 4:** Summary of the CHELPG Charges (in au) Obtained from Gaussian16 for Each Residue of the QM Region for the Stationary Structures

residue	ES complex	INT1	INT2	INT3	INT4	product
ASP234	–0.78	–0.75	–0.73	–0.79	–0.78	–0.75
ASP232	–0.69	–0.67	–0.67	–0.67	–0.69	–0.68
HIS141	0.02	0.06	0.09	0.08	0.03	0.02
ASP128	–0.75	–0.67	–0.66	–0.53	–0.70	–0.73
HIS126	0.11	0.14	0.16	0.16	0.11	0.18
ASP124	–0.63	–0.71	–0.70	–0.69	–0.68	–0.66
HIS101	0.18	0.21	0.16	0.21	0.22	0.20
Mn_B_	1.18	1.12	1.14	1.12	1.17	1.18
Mn_A_	1.16	1.08	1.09	1.07	1.17	1.19
OH	–0.51	–0.12	–0.12	–0.25	–0.54	–0.32
l-ARG^+^	0.71	0.31	0.25	0.22	0.69	0.37

**Figure 9:**
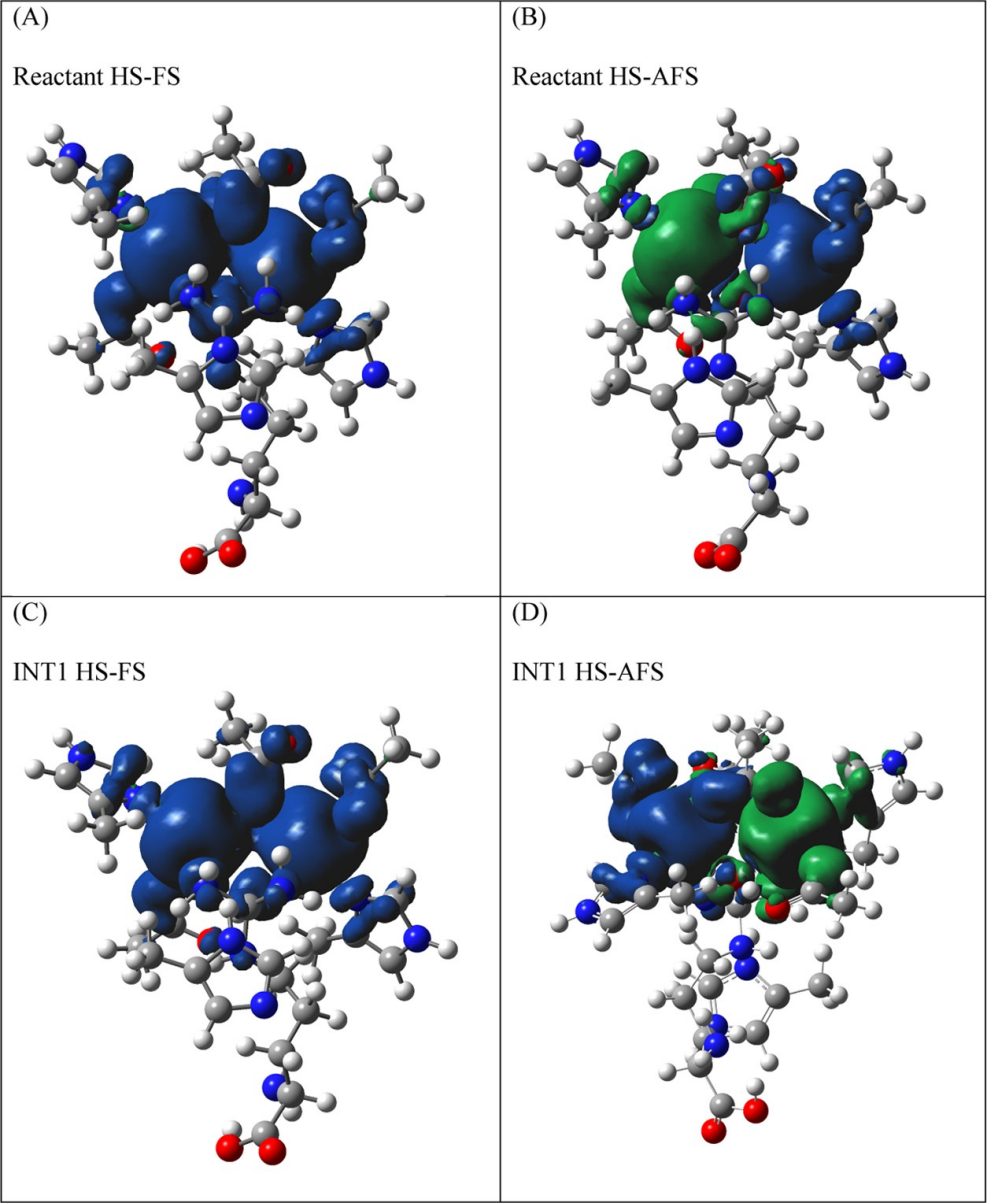
Spin density plot of the QM region of the reactant and INT1 for their HS-FS and HS-AFS states. (A) Reactant HS-FS; (B) reactant HS-AFS; (C) INT1 HS-FS; and (D) INT1 HS-AFS.

Analyzing the *S*_αβ_ value, it was noticed that for the ES complex, there was a significant orbital overlap between the spin on the Mn^2+^ centers compared with the other TSs and intermediates. From Figure 9, interestingly, it can be noted that the hydroxide ion has both α and β spin densities and it is coupled with the spin densities of the two Mn(II) ions. The spin density plot (Figure 9) of the ES complex and INT1 indicates an antiferromagnetic superexchange pathway taking place in the ES complex via the bridged hydroxide ion.

**Figure 10:**
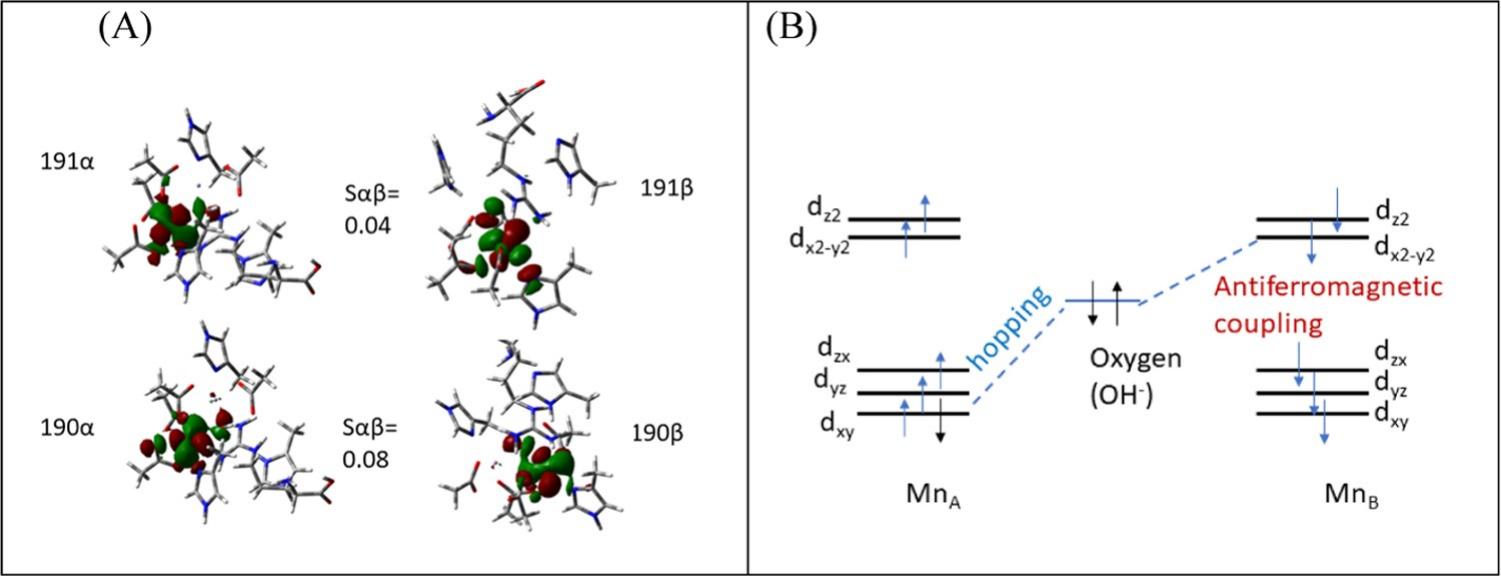
(A) Two dominant antiferromagnetic coupling molecular orbitals for the broken-symmetry ES complex. (B) Schematic mechanism of atomic orbitals participating in the antiferromagnetic superexchange interaction between the two manganese atoms.

In INT1, due to the transfer of the hydroxide ion to the substrate carbon, there is a significant reduction in antiferromagnetic superexchange, which leads to a decrease in the *S*_αβ_ value between molecular orbitals (MO)s and is also reflected in the *J*_ab_ value. In TS5 and the product, there is no significant overlap in MOs compared with the ES complex. However, the increased value of *J*_ab_ is due to the greater stabilization of the HS-AFS state compared with the high-spin state in the product. An antiferromagnetic superexchange interaction occurs between the two manganese centers. The unpaired electron on the p-orbital of OH couples with the electron of one of the five d orbitals (dz^2^) at Mn2. The two electrons localized at the Mn^2+^ ions are coupled by superexchange antiferromagnetically. A previous report^38^ showed that exchange and superexchange interactions can dictate the catalytic mechanism and activity of metalloenzymes by influencing the electron transfer in the catalytic site. In metalloenzymes, exchange and superexchange interactions are important for the stabilization of the catalytic site. For instance, two identical spins on the two Fe centers and their orbital interactions with the bridging ligand in [4Fe–4S]-containing metalloenzymes would lead to destabilization, whereas the same Fe centers with opposite spins stabilize the interactions through the superexchange mechanism, which is the interaction of singly occupied orbitals of metals with the doubly occupied orbital of the bridging ligand. Similarly, in HARGI, the superexchange interaction of the doubly occupied orbital of the hydroxide ion and the bridging ligand with singly occupied orbitals of Mn ions stabilizes the broken-symmetry state of the catalytic site. Overall, it is evident that the hydroxide ion plays an important role in the antiferromagnetic coupling of the two Mn(II) ions. Two dominant antiferromagnetic coupling molecular orbitals for the broken-symmetry ES complex are shown in Figure 10.

### 3.4 Charge Transfer

The charges from electrostatic potentials using a grid-based method (CHELPG) were used to calculate the atomic charges on the manganese center, hydroxide moiety, l-arginine, and amino acid residues surrounding the l-arginine ligand for all of the intermediates of the high-spin state along the predicted mechanistic pathway (Table 4).^34^ The Mn_1_ and Mn_2_ centers in the ES complex possess charges of 1.16 and 1.18 au, respectively. Throughout the potential energy pathway for the intermediates INT1, INT2, INT3, and INT4 and the product, the charge on the Mn_1_ and Mn_2_ centers remains almost constant and unchanged. Hence, no charge transfer occurs from the manganese center to the l-arginine ligand or surrounding amino acid residues in the reaction pathway for the hydrolysis of l-Arg by human arginase. The negative charge on the oxygen center of the hydroxide moiety (OH) gets reduced in INT1 after getting transferred to the carbon center of the guanidine group of l-ARG from the bridging position between the two Mn centers in the ES complex. Atomic charges on each residue are given in Table S3. A slight increase in the negative charge on the oxygen atom of the OH center occurs at INT-3, where proton transfer to Asp128 (OD1) takes place. In INT4, the transfer of the proton to the NE center of the guanidine group in l-ARG leads to a reduction in the positive charge of the same proton; hence, the overall charge of OD1 is more negative in INT4. The charge on most of the amino acid residues (Asp234, Asp232, His141, His126, Asp124, His101) surrounding the l-ARG ligand remains constant throughout the potential energy surface. Only a reduction in the negative charge on Asp128 is noticed at INT3 due to the transfer of a proton from the hydroxyl group-bound l-ARG ligand. The positive charge on the Arg^+^ ligand gets reduced from the ES complex to INT1 due to the transfer of the negatively charged hydroxyl center to ARG^+^. Again, an increase in the positive charge is noticed in l-ARG^+^ in INT4 due to the transfer of a proton to the NE center of its guanidino group and reduction of the negative charge on the nitrogen center. Dominant antiferromagnetic coupling molecular orbitals for broken-symmetry-optimized structures of INT1, INT2, INT3, INT4, and the product in the high-spin potential energy surface are given in Figures S5–S7.

## 4 Conclusions

In essence, we employed classical molecular dynamics simulations coupled with quantum mechanics and molecular mechanics (QM/MM) to explore the hydrolysis reaction mechanism of human arginase I with antiferromagnetic and ferromagnetic coupling between two Mn(II) ions at the catalytic site. In our study, we considered the hydroxide ion as a nucleophile and the guanidino group of the substrate as a positively charged species. We presumed that the presence of the hydroxide ion is crucial to attack the electrophilic site of the substrate and for ferromagnetic and anti-ferromagnetic coupling between Mn ions. In addition, the orientation of the hydroxide ion toward Asp128 and its interaction with the guanidine group of the substrate are important for the initiation of the catalytic mechanism of HARGI.

Our QM/MM calculations showed that the ES complex of the broken-symmetry antiferromagnetic coupled state is more stabilized (0.39 kcal/mol) than that of the high-spin ferromagnetically coupled state. Moreover, no significant changes were observed in the substrate orientation toward Mn(II) ions, and also, variations in the distance between the two Mn(II) ions are small. We also observed that the ES complex for the triplet state is destabilized (∼104 kcal/mol) compared with the high-spin broken-symmetry (BS) ES complex. Similarly, the broken-symmetry singlet low-spin state ES complex is also destabilized (136 kcal/mol) compared with the high-spin ES (BS) complex. Our calculations revealed that low-spin ferromagnetic and antiferromagnetic states are not stable compared with the high-spin states, which showed that the high-spin BS (antiferromagnetic) state is more stable compared with all other states.

Our investigation shows that the mechanistic paths for the high-spin and low-spin states of the enzyme are similar, which indicates that the reaction mechanism is independent of the spin state of the enzyme. Moreover, the potential energy surface of the high-spin antiferromagnetic state is energetically the most favorable as compared with the high-spin ferromagnetically coupled state. We found that the cleavage of the NE–CZ bond and the formation of the l-ornithine product is the rate-determining step with the highest TS barriers, which is in agreement with a previous study.^15^ From our QM/MM calculations, it is confirmed that the high-spin broken-symmetry state (BS) of human arginase is more favored than its HS state. It is difficult to predict whether only the antiferromagnetically coupled BS spin state exists or a mixture of both the HS and HS-BS spin states of the enzyme undergoes the catalytic reaction. Our spin density analysis showed that the spin on the hydroxide ion is coupled with an opposite spin on two Mn(II) ions in the reactant in the high-spin broken-symmetry state. This shows the antiferromagnetic superexchange interaction between two Mn(II) ions. The calculated coupling constant (*J*_ab_) values also evidence that the antiferromagnetic coupling is stronger than the ferromagnetic coupling.

Our detailed understanding of the reaction mechanism of human arginase I will be useful for further designing better inhibitors for various disease conditions.

## Supporting Information Available

MD simulations, various geometric parameters calculated throughout MD simulations, geometrical parameters calculated for the broken-symmetry singlet low-spin state, calculated Mulliken spin densities for all possible ferromagnetic and antiferromagnetic states of manganese ion, INT structures along with the product structure, CHELPG charges (in au) obtained from gaussian16 for each atom of each residue in the QM region, two dominant antiferromagnetic coupling molecular orbitals for broken-symmetry-optimized structures of INT1, INT2, INT3, INT4, and the product in the high-spin potential energy surface (PDF)

## Abbreviations

HARGI: human arginase I
MD: molecular dynamics
QM/MM: quantum mechanics/molecular mechanics
INT: intermediates
TS: transition state
ES: enzyme–substrate
RARGI: rat liver arginases
EPR: electronic paramagnetic resonance
