# Supplementary material for: QM/MM Simulations for the Broken-Symmetry Catalytic Reaction Mechanism of Human Arginase I
Source: ACS Omega. 2022 Aug 30;7(36):32536–48. doi: 10.1021/acsomega.2c04116 (PMC9475637; doi:10.1021/acsomega.2c04116)
Supplement: Supplementary file 1 — ao2c04116_si_001.pdf [file ao2c04116_si_001.pdf]

QM/MM simulations for broken symmetry catalytic reaction mechanism for Human Arginase I

*Sathish kumar Mudedla[1], Boyli Ghosh[2], Gaurao V. Dhoke[2], Se Kyu Oh[3] and Sangwook Wu[1,4,]**

[1]R&D Center, Pharmcadd, 12F, 331, Jungang-daero, Dong-gu, Busan, Republic of Korea

[2]R&D Center, Pharmcadd, Hyderabad, India

[3]KYNOGEN Co., Suwon 16229, Republic of Korea

[4]Department of Physics, Pukyong National University, Busan 48513, Republic of Korea

*Corresponding author:

*Sangwook Wu, CEO, R&D center, Pharmcadd, 331, Jungang-daero, Dong-gu, Busan, Republic of Korea, phone: 82-51-731-5688, fax: 82-51-731-5689,

Email: s.wu@pharmcadd.com

Supporting Information

Table of Contents

1. Calculated RMSD and geometrical parameters throughout 100 ns of MD simulations for human arginase I………...Figure S1
2. Various Geometric Parameters Calculated Throughout MD Simulations of Human Arginase 1.........Table S1
3. The geometrical parameters were calculated for the broken symmetry Singlet low spin state……Table S2
4. Spin densities of high spin ferromagnetic and high spin antiferromagnetic states……. Figure S2
5. INTs structures along with product structure of the mechanism of hydrolysis of human arginase……. Figure S3
6. The potential energy profiles of high spin ferromagnetic and high spin antiferromagnetic states……Figure S4
7. CHELPG charges (in a.u) obtained from gaussian16 for each atom of each residue of the QM region for the stationary structures……Table S3
8. Two dominant antiferromagnetic coupling molecular orbitals for broken symmetry optimised structure of INT1 and INT2 in the high spin potential energy surface……... Figure S5
9. Two dominant antiferromagnetic coupling molecular orbitals for broken symmetry optimised structure of INT3 and INT4 in the high spin potential energy surface……...Figure S6
10. Two dominant antiferromagnetic coupling molecular orbitals for broken symmetry optimised structure of Product in the high spin potential energy surface………Figure S7

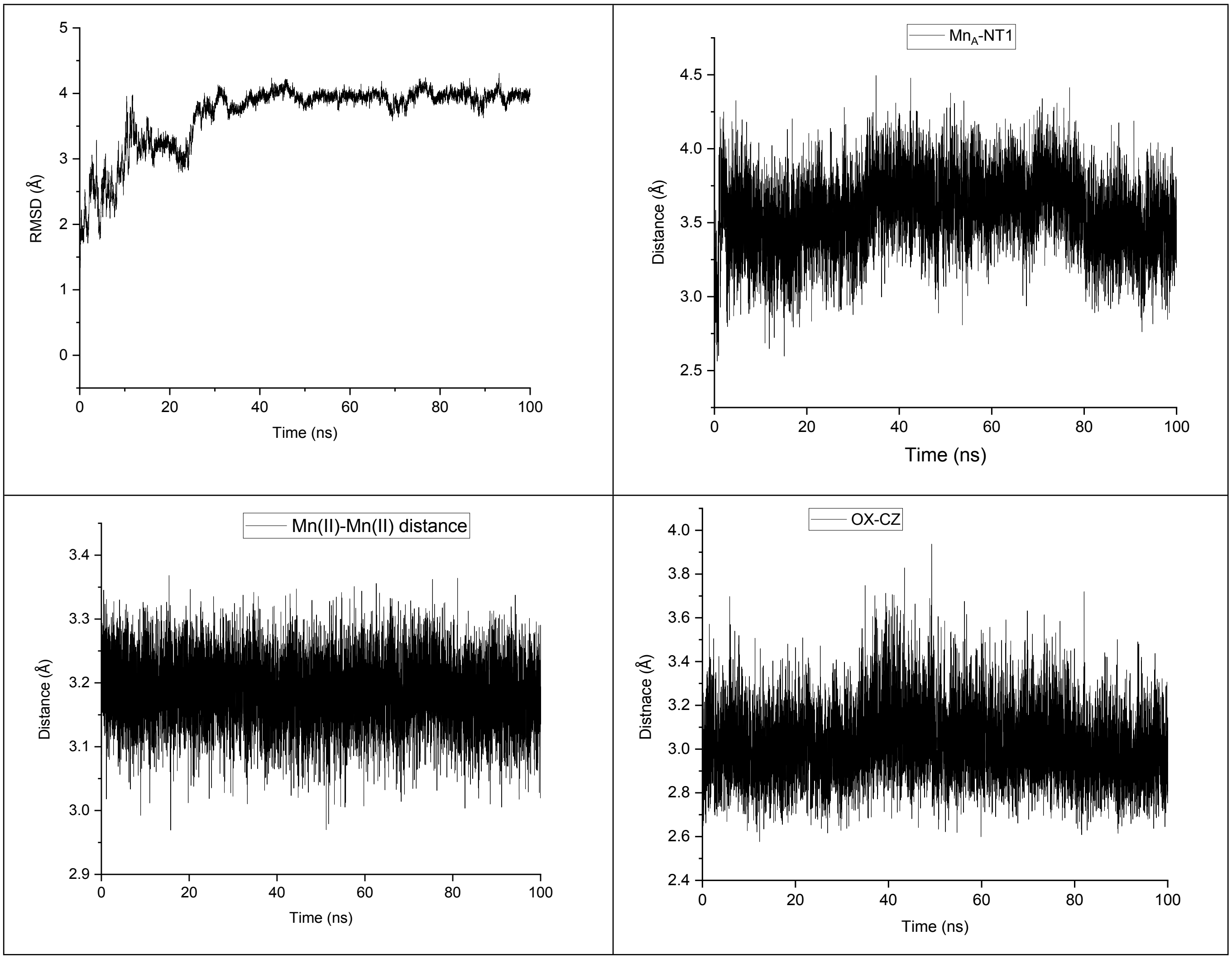

Figure S1. RMSD values and distance during 100 ns of MD simulations for human arginase I. Mn(II) (manganese ion), NT1 (one of the nitrogen atoms in guanidine group of L-arginine), OX(oxygen in hydroxide ion), CZ (carbon in guanidine group of L-arginine).

Table S1. Various Geometric Parameters Calculated Throughout MD Simulations of Human Arginase 1.

Parameters	Distances (Å) in Final MD Structure
Mn_1-Mn_2	3.19
OX-CZ	2.81
NT1-Mn_1	3.25
ASP128:OD1-HX	2.81
HX-OX	0.98
HX-NE	2.48
ASP128:OD1- NE	2.81
Mn_1-OX	1.83
Mn_2-OX	1.81
NE-CZ	1.29

Table S2. The Geometrical Parameters were Calculated for The Broken Symmetry Singlet Low Spin State. Distances are in Å.

**Coordinates**	**Reactant (Triplet)**	**Reactant (High-spin-ferromagnetic state)**	**Reactant (low spin broken symmetry)**
d(OX-CZ)	2.17	2.39	2.40
d(NT1-Mn_1)	2.33	2.74	2.76
d(ASP128:OD1-HX)	1.71	1.71	1.70
d(HX- OX)	0.98	0.98	0.98
d(HX-NE)	2.61	2.73	2.74
d(ASP128:OD1- NE)	3.04	3.38	3.39
D(Mn_1-Mn_2)	3.13	3.15	3.14

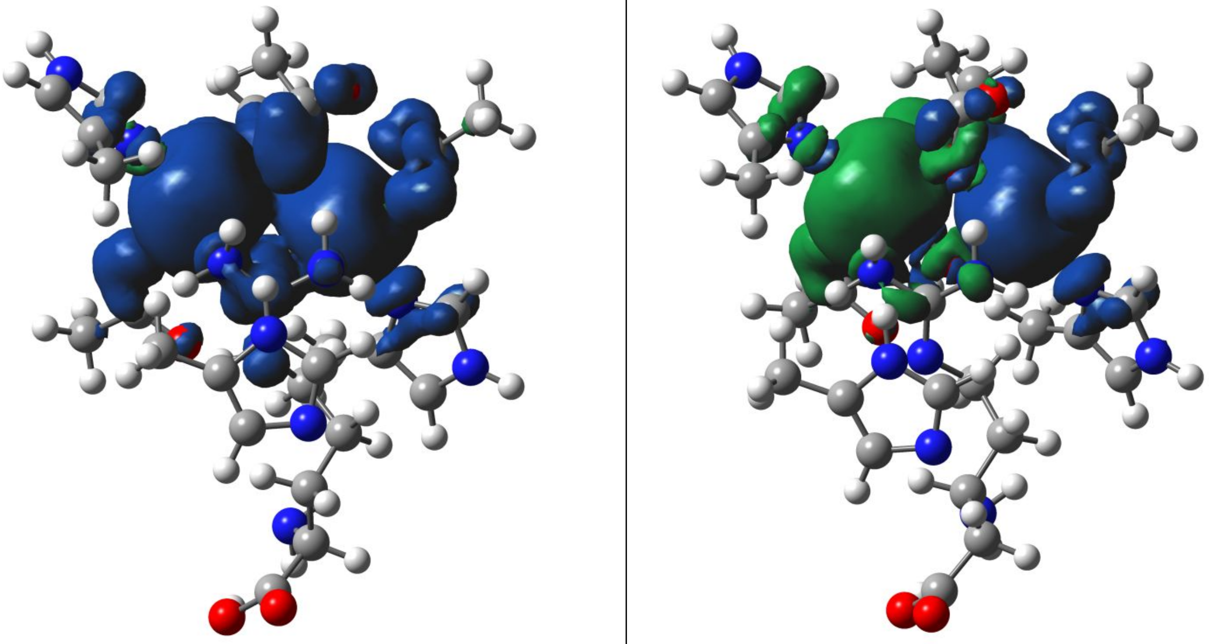

HS-FS

HS-AFS

Figure S2. Spin Densities of High Spin Ferromagnetic State and High Spin Antiferromagnetic State. Blue colour indicates up spin of electrons and green colour denotes down spin of electrons.

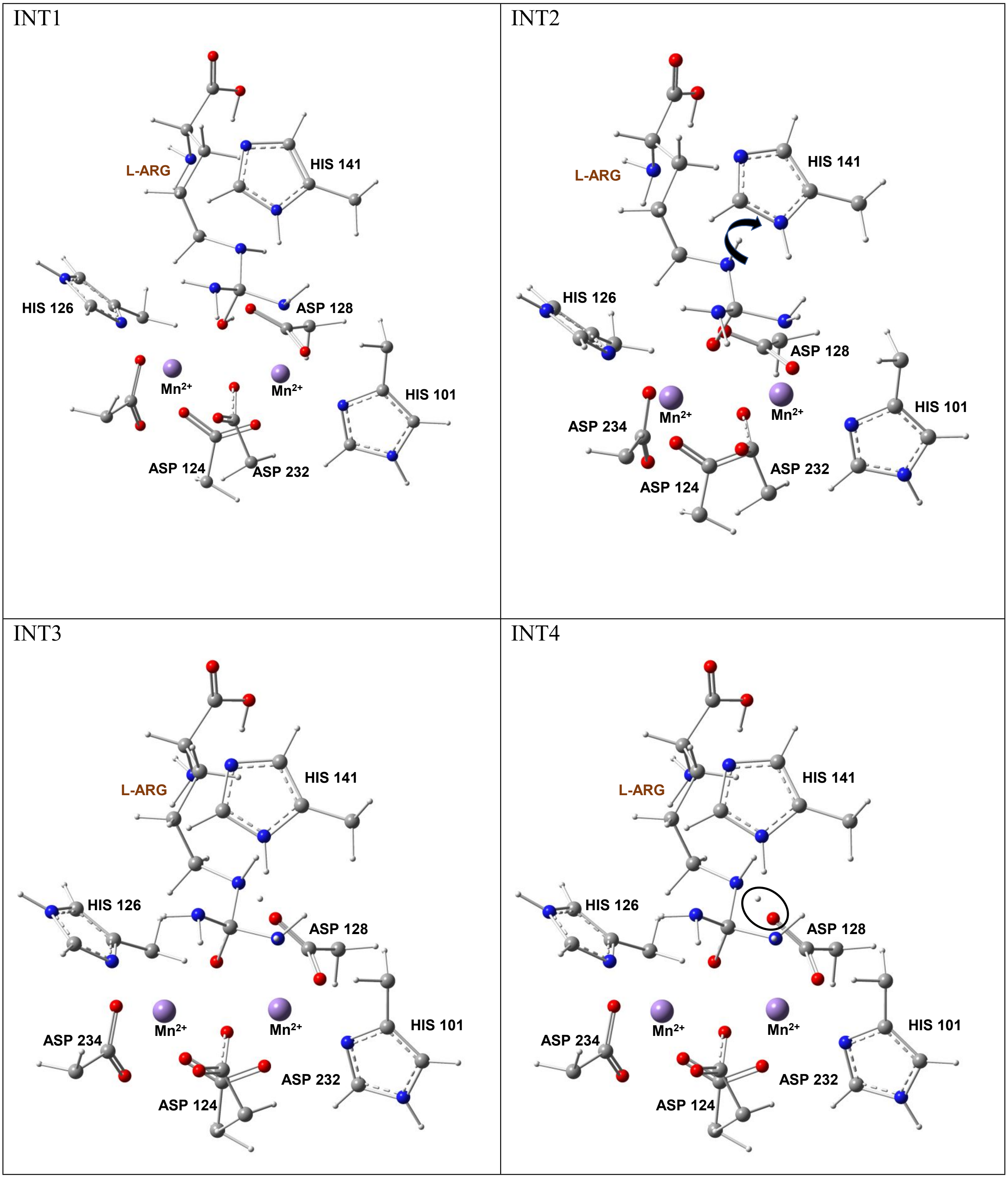
INT1
L-ARG
HIS 141
HIS 126
ASP 128
Mn2+
Mn2+
HIS 101
ASP 124
ASP 232
INT2
L-ARG
HIS 141
HIS 126
ASP 128
Mn2+
Mn2+
HIS 101
ASP 234
ASP 232
ASP 124
INT3
L-ARG
HIS 141
HIS 126
ASP 128
Mn2+
Mn2+
HIS 101
ASP 234
ASP 232
ASP 124
INT4
L-ARG
HIS 141
HIS 126
ASP 128
Mn2+
Mn2+
HIS 101
ASP 234
ASP 232
ASP 124

Product

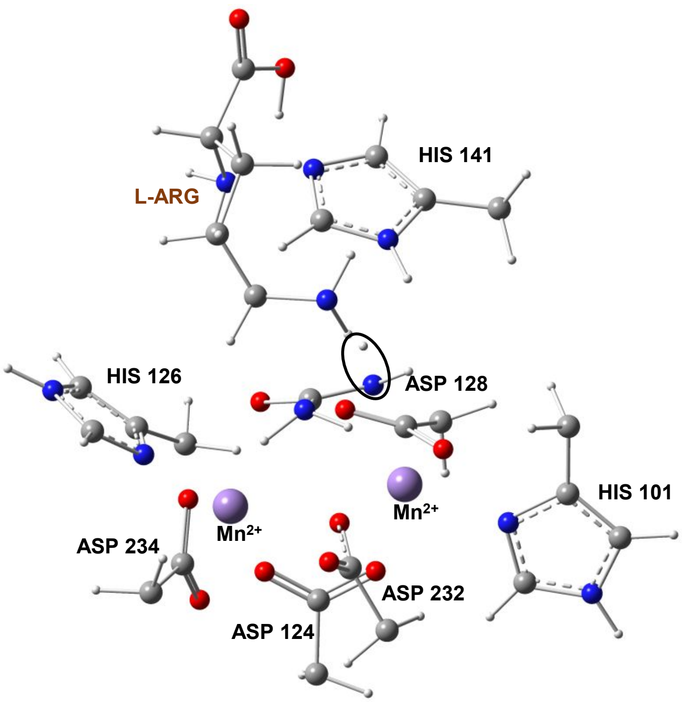

Figure S3. INTs Structures Along with Product Structure of The Mechanism of Hydrolysis of Human Arginase. Hydrogen Involved in the Reaction is Initially Belongs to the OH ion in INT3, INT4 and Product.

(A)

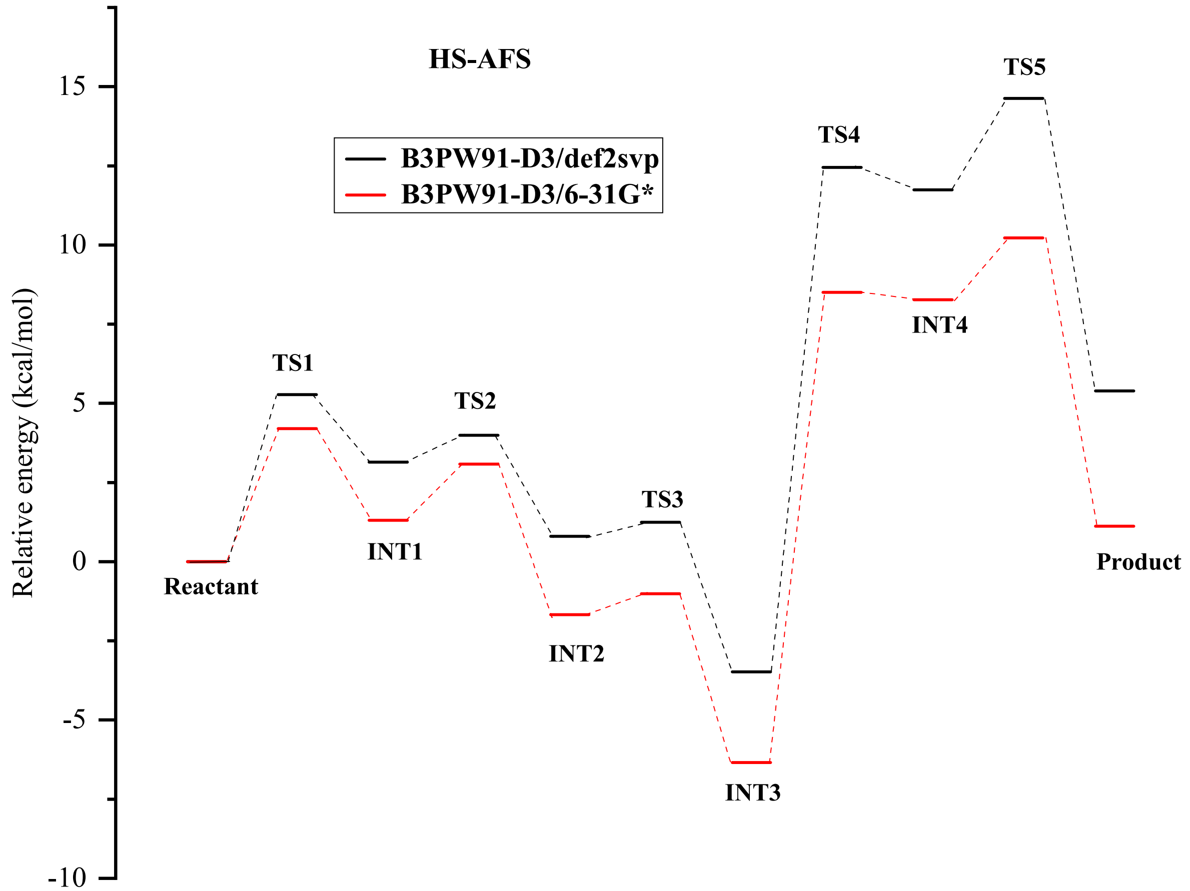

HS-AFS
B3PW91-D3/def2svp
B3PW91-D3/6-31G*
Relative energy (kcal/mol)
15
10
5
0
-5
-10
TS1
TS2
TS3
TS4
TS5
Reactant
INT1
INT2
INT3
INT4
Product

(B)

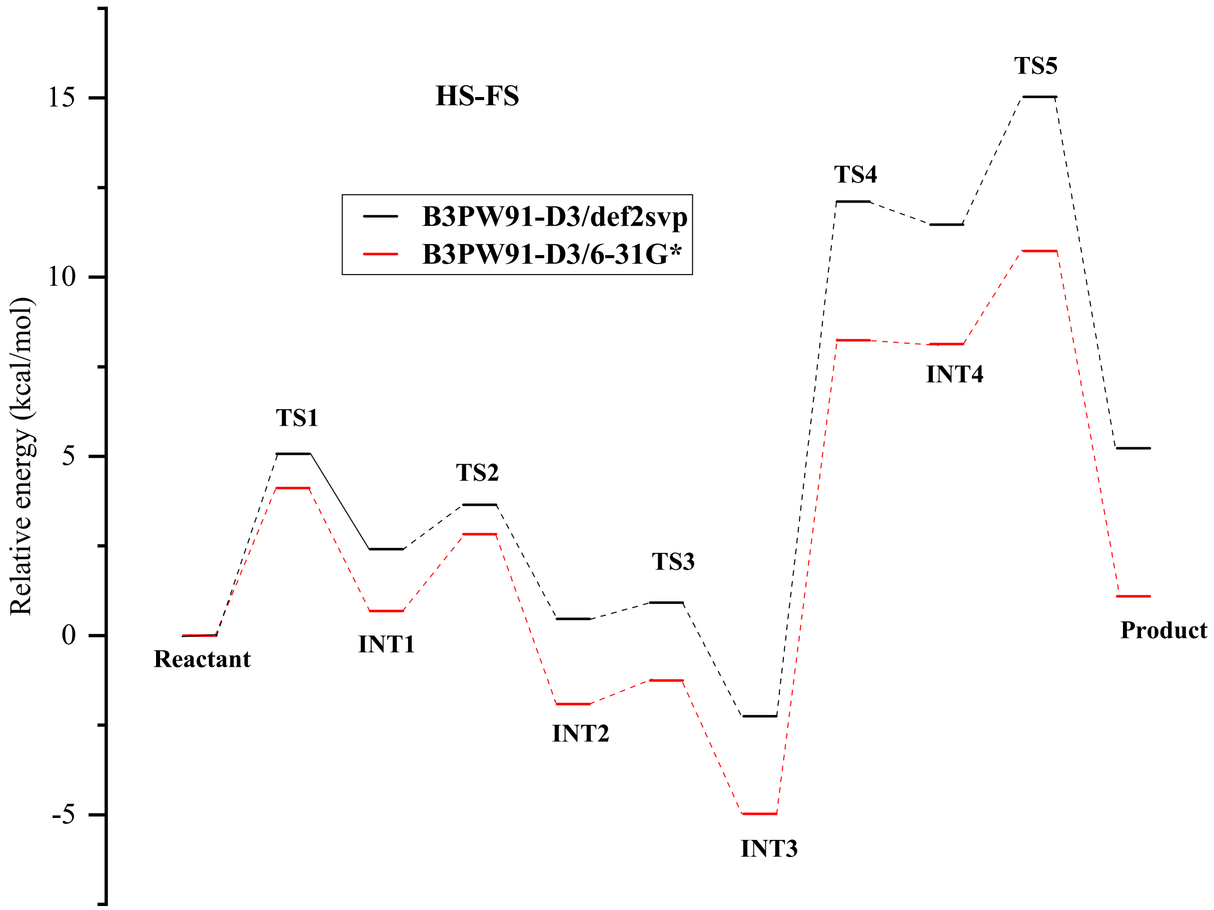

HS-FS
B3PW91-D3/def2svp
B3PW91-D3/6-31G*
Relative energy (kcal/mol)
15
10
5
0
-5
Reactant
TS1
INT1
TS2
INT2
TS3
INT3
TS4
INT4
TS5
Product

(C)

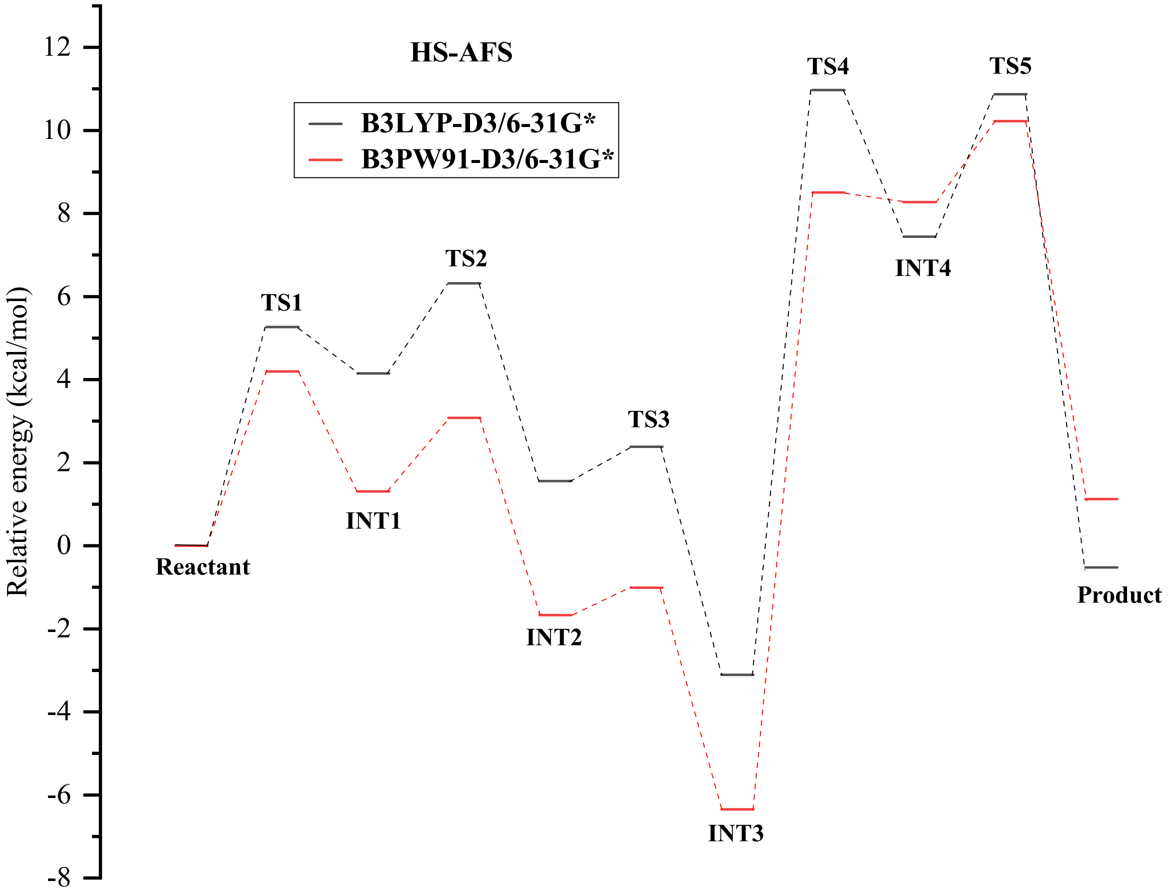
HS-AFS
B3LYP-D3/6-31G*
B3PW91-D3/6-31G*
Relative energy (kcal/mol)
12
10
8
6
4
2
0
-2
-4
-6
-8
Reactant
TS1
INT1
TS2
INT2
TS3
INT3
TS4
INT4
TS5
Product

(D)

Residue	Atom Symbol	ES-Complex	INT1	INT2	INT3	INT4	Product
ASP 234	CB	-0.31365	-0.26309	-0.23997	-0.25891	-0.25922	-0.28387

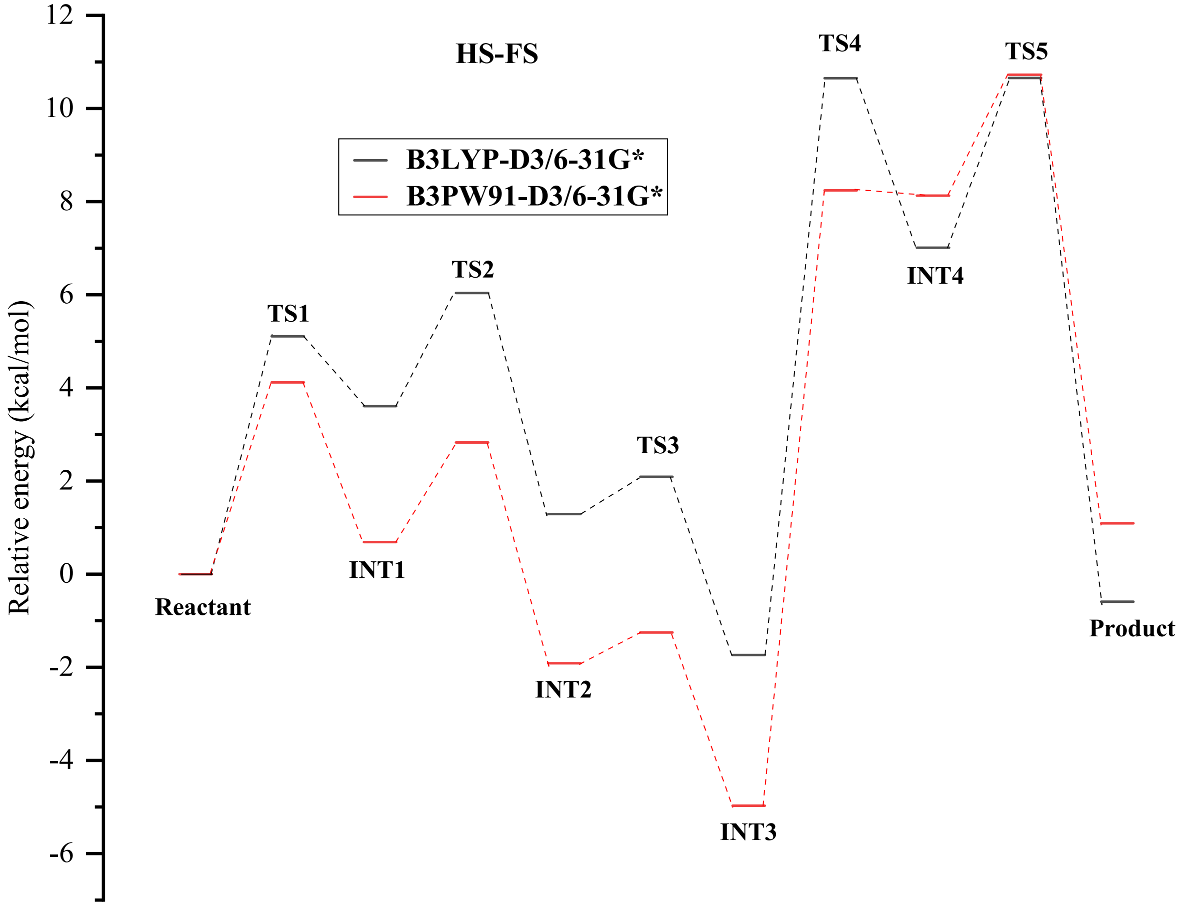

Figure S4. The potential energy profiles for HS-FS and HS-AFS at B3PW91-D3/6-31G*, B3PW91-D3/def2svp and B3LYP-D3/6-31G* levels of theory. (A) HS-AFS (B) HS-FS (C) HS-AFS and (D) HS-FS.

	HB1	0.096048	0.088628	0.081011	0.085248	0.083018	0.083561
	HB2	0.061774	0.052081	0.046032	0.048707	0.048276	0.065378
	CG	0.850839	0.840185	0.833979	0.83947	0.858867	0.869135
	OD1	-0.76521	-0.74918	-0.75109	-0.7542	-0.77581	-0.77624
	OD2	-0.71192	-0.7097	-0.71093	-0.70623	-0.71857	-0.7126
	H	0.135256	0.035087	0.047836	0.059647	0.074018	0.080661
ASP 232	CT	-0.36277	-0.28008	-0.27354	-0.25825	-0.32134	-0.26698
	HC	0.148236	0.126481	0.123995	0.11915	0.131261	0.124843
	HC	0.014996	-0.01086	-0.00593	-0.01065	0.013537	-0.01303
	C	0.825187	0.784974	0.785883	0.790441	0.850139	0.741851
	OD1	-0.60931	-0.57612	-0.57772	-0.58024	-0.60644	-0.59287
	OD2	-0.86453	-0.84463	-0.86002	-0.85126	-0.91959	-0.81731
	H	0.059339	0.043401	0.068842	0.069466	0.065092	0.070208
HIS 141	CB	-0.25549	-0.09609	-0.12153	-0.17712	-0.15911	-0.21262
	HB1	0.105022	0.05155	0.059913	0.072041	0.073437	0.073614
	HB2	0.069012	0.037989	0.048823	0.06815	0.058027	0.069428
	CG	0.10281	0.04658	0.05301	0.056565	0.055511	0.115028
	ND1	-0.29977	-0.27506	-0.2399	-0.22315	-0.20697	-0.29026
	HD1	0.278477	0.244008	0.239616	0.236374	0.219353	0.273536
	CE1	0.164261	0.216614	0.174837	0.174986	0.169993	0.151988
	HE1	0.089516	0.056564	0.074907	0.07632	0.07432	0.087928
	NE2	-0.3967	-0.45741	-0.43781	-0.45165	-0.44034	-0.39182
	CD1	-0.04771	0.002816	0.005938	0.008373	0.000803	-0.01197
	HD2	0.130672	0.107935	0.111702	0.11025	0.111788	0.096952
	H	0.087703	0.126611	0.125509	0.128195	0.072311	0.055585

Table S3. CHELPG Charges (in a.u) Obtained from Gaussian16 for Each Atom of Each Residue of the QM Region for the Stationary Structures.

ASP 128	CB	-0.3586	-0.35472	-0.34877	-0.33027	-0.33539	-0.33212
	HB1	0.08136	0.081695	0.078775	0.089277	0.076206	0.071702
	HB2	0.104637	0.107387	0.100715	0.111141	0.094596	0.092693

	CG	0.843171	0.858317	0.864276	0.834122	0.884162	0.869962
	OD1	-0.697	-0.69334	-0.69159	-0.61565	-0.7053	-0.69965
	OD2	-0.79792	-0.75119	-0.75256	-0.66298	-0.75989	-0.78386
	H	0.067862	0.07582	0.082042	0.036994	0.043806	0.041783
HIS 126	CB	-0.37793	-0.24009	-0.20386	-0.2222	-0.21897	-0.19319
	HB1	0.106481	0.068251	0.054668	0.05341	0.046791	0.053436
	HB2	0.136202	0.092284	0.090611	0.088604	0.101854	0.10314
	CG	0.307866	0.274008	0.261764	0.28056	0.289793	0.291761
	ND1	-0.37892	-0.35911	-0.37254	-0.34333	-0.37625	-0.40443
	CE1	0.049091	0.04648	0.070292	0.052099	0.063082	0.058327
	HE1	0.135282	0.13341	0.127447	0.134882	0.133827	0.131233
	NE2	-0.17287	-0.16886	-0.19283	-0.19188	-0.19416	-0.1468
	HE2	0.308661	0.306542	0.311265	0.310528	0.309066	0.296321
	CD2	-0.27826	-0.24752	-0.23444	-0.24456	-0.24996	-0.27792
	HD2	0.170316	0.162597	0.160097	0.160552	0.155538	0.16524
	H	0.114011	0.081151	0.08555	0.085084	0.052503	0.100242
ASP 124	CB	-0.48642	-0.46485	-0.46334	-0.45603	-0.45878	-0.47387
	HB1	0.122372	0.115544	0.114817	0.110827	0.112167	0.119175
	HB2	0.101652	0.097951	0.096963	0.093924	0.097974	0.103432
	CG	0.937103	0.998357	1.005943	0.978691	0.997872	1.019991
	OD1	-0.73046	-0.76042	-0.76332	-0.74093	-0.74558	-0.76721
	OD2	-0.70971	-0.73679	-0.74131	-0.73494	-0.75913	-0.74781
	H	0.135256	0.035087	0.047836	0.059647	0.074018	0.080661
HIS 101	CB	-0.32141	-0.215	-0.23701	-0.24916	-0.21164	-0.29173
	HB1	0.08353	0.064706	0.07461	0.074142	0.060262	0.090136
	HB2	0.091992	0.060508	0.063822	0.071069	0.06095	0.064664
	CG	0.225374	0.203716	0.213271	0.229122	0.225828	0.255233
	ND1	-0.35109	-0.33696	-0.33621	-0.34662	-0.35752	-0.39774
	CE1	0.254958	0.218942	0.213565	0.219738	0.219737	0.259318
	HE1	0.097042	0.106936	0.106465	0.104907	0.101622	0.092789

	NE2	-0.33681	-0.30957	-0.30391	-0.31123	-0.29675	-0.31354
	HE2	0.355726	0.345215	0.344052	0.345723	0.338746	0.344648
	CD2	-0.19773	-0.17158	-0.18101	-0.18675	-0.20323	-0.1867
	HD2	0.172994	0.16227	0.164682	0.164314	0.166654	0.164166
	H	0.110006	0.080154	0.039843	0.09415	0.119974	0.122387
**Mn_2**	**Mn**	1.18	1.12	1.14	1.12	1.17	1.18
**Mn_1**	**Mn**	1.16	1.08	1.09	1.07	1.17	1.19
**OH**	**O**	-0.89028	-0.43651	-0.4807	-0.64486	-0.66752	-0.56014
	**H**	0.371527	0.311475	0.359268	0.395588	0.122673	0.244007
**L-Arg+**	**HD1**	-0.04704	-0.13157	-0.09601	-0.14744	0.062383	0.061363
	**HA1**	0.015845	0.001303	-0.00548	-0.00043	0.005235	0.005206
	**HG2**	0.089767	0.080727	0.057573	0.048599	0.061555	0.038558
	**HB1**	-0.14492	-0.12621	-0.12389	-0.11199	-0.12059	-0.10369
	**HB2**	-0.05444	-0.08321	-0.06831	-0.06462	-0.08114	0.009296
	**HD2**	0.017868	-0.04392	0.001368	-0.05067	0.103039	0.006618
	**HG1**	0.026912	-0.01543	-0.01038	-0.02803	0.015972	-0.01902
	**CG**	-0.40749	-0.29336	-0.23893	-0.19757	-0.20408	-0.12
	**CB**	0.556216	0.493238	0.441451	0.40216	0.459989	0.31385
	**CA**	0.02499	0.068914	0.10578	0.094579	0.070274	0.095013
	**N**	-0.92441	-0.89238	-0.88103	-0.87655	-0.9316	-0.90202
	**NH1**	0.381419	0.354449	0.350652	0.349305	0.373547	0.355624
	**NH2**	0.329647	0.334117	0.335041	0.333257	0.341993	0.334092
	**NH3**	0.361084	0.341116	0.334703	0.335343	0.368337	0.353878
	**C**	0.58565	0.613249	0.604527	0.618243	0.628572	0.612842
	**O1**	-0.50489	-0.50942	-0.50634	-0.51046	-0.516	-0.51564
	**O2**	-0.4746	-0.50485	-0.5075	-0.51047	-0.50067	-0.51093
	**CD**	0.441522	0.509358	0.429427	0.565356	-0.11238	0.092772
	**NE**	-0.72525	-0.78937	-0.95913	-1.04485	0.163798	-0.45896
	**HE**	0.33873	0.29629	0.358881	0.365894	0.073834	0.155241
	**CZ**	1.033348	0.990797	1.062519	1.172248	0.931725	0.936397
	**NT1**	-0.88442	-0.89083	-0.97586	-1.03247	-0.88226	-0.90823

**NH1**	0.427794	0.376184	0.383996	0.380314	0.324267	0.450887
**NH2**	0.407582	0.32651	0.356293	0.360875	0.325871	0.371264
**NT2**	-1.02888	-0.96356	-0.93918	-0.94932	-1.03419	-0.94104
**NH1**	0.412493	0.365472	0.350068	0.340527	0.353377	0.279709
**NH2**	0.452186	0.402881	0.389446	0.377866	0.412987	0.383374

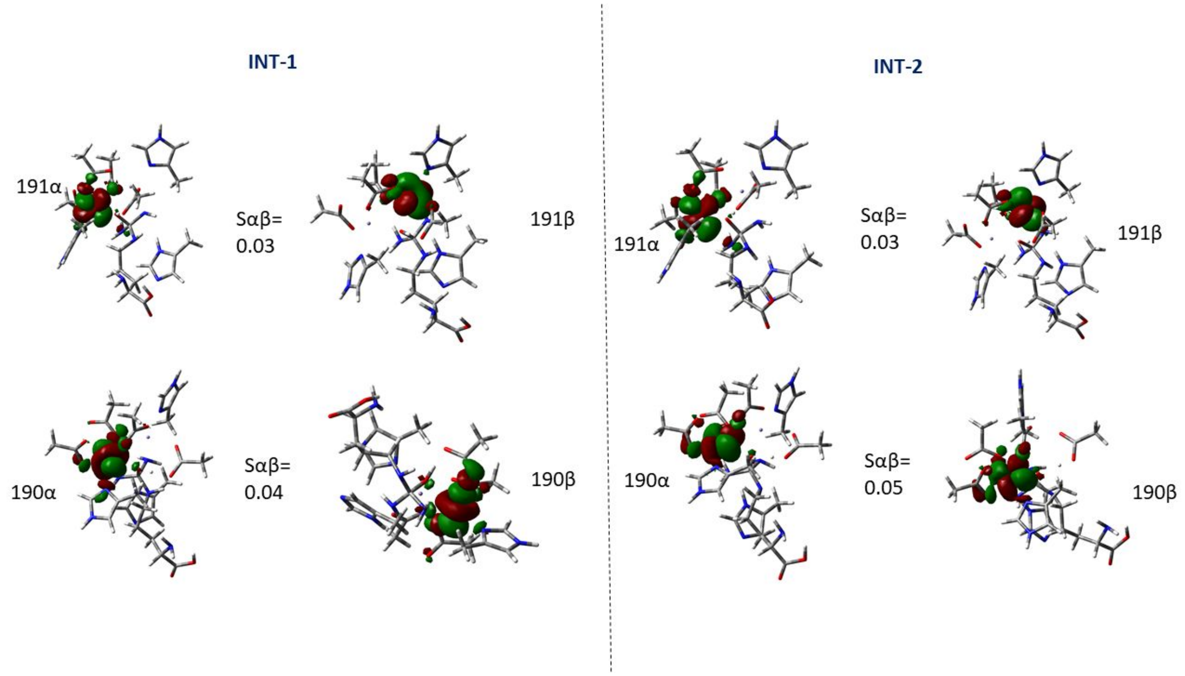

Figure S5. Two Dominant Antiferromagnetic Coupling Molecular Orbitals for Broken Symmetry Optimised Structure of INT1 and INT2 in the High Spin Potential Energy Surface.

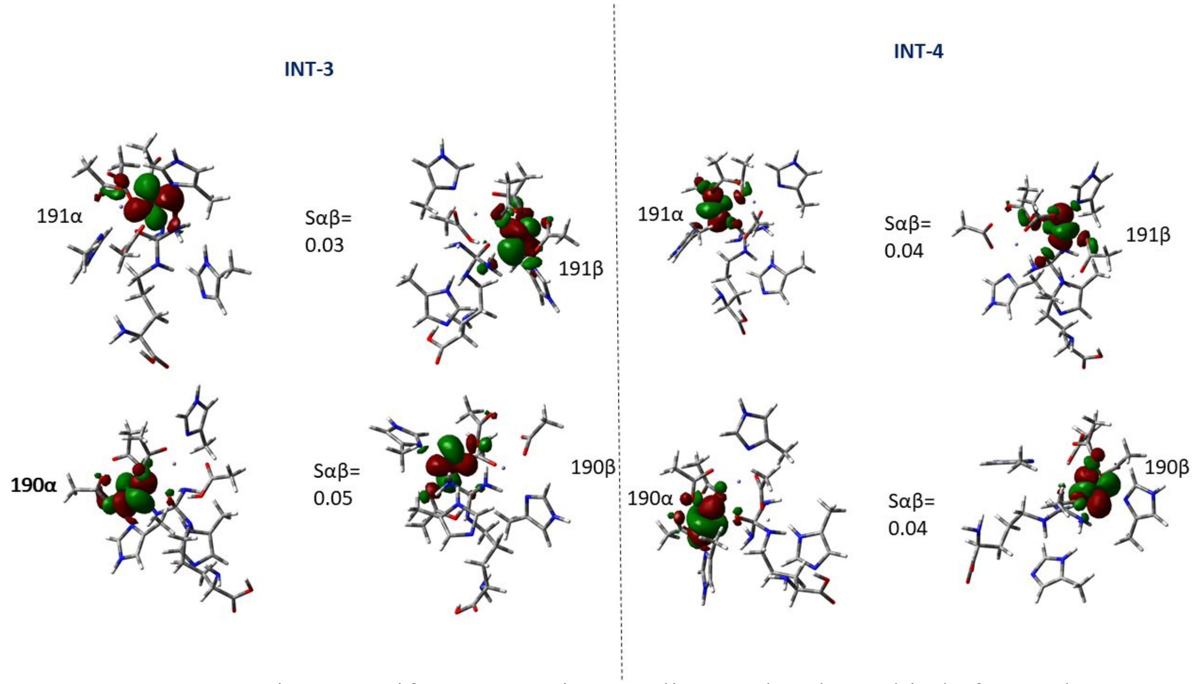

Figure S6. Two Dominant Antiferromagnetic Coupling Molecular Orbitals for Broken Symmetry Optimised Structure of INT3 and INT4 in the High Spin Potential Energy Surface.

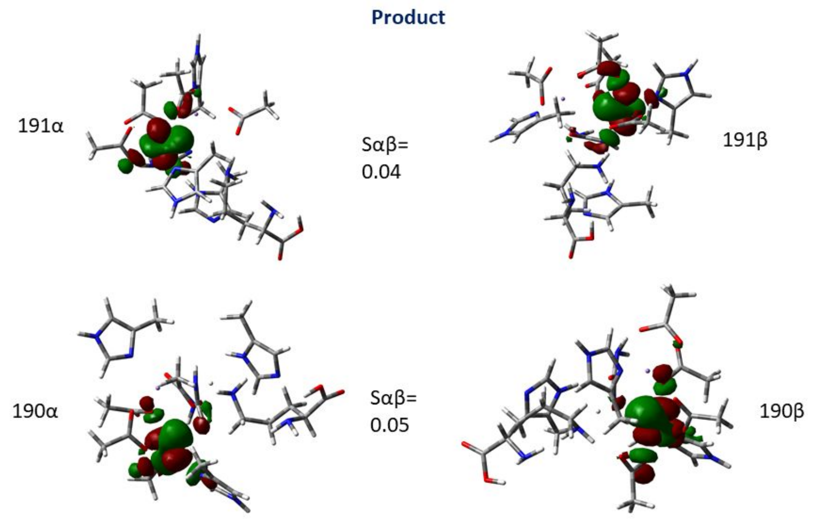

Figure S7. Two Dominant Antiferromagnetic Coupling Molecular Orbitals for Broken Symmetry Optimised Structure of Product in the High Spin Potential Energy Surface.
